# Endocytic recycling *via* the TGN underlies the polarized hyphal mode of life

**DOI:** 10.1371/journal.pgen.1007291

**Published:** 2018-04-02

**Authors:** Miguel Hernández-González, Ignacio Bravo-Plaza, Mario Pinar, Vivian de los Ríos, Herbert N. Arst, Miguel A. Peñalva

**Affiliations:** 1 Department of Cellular and Molecular Biology and Intradepartmental WhiteBiotech Unit, Centro de Investigaciones Biológicas del Consejo Superior de Investigaciones Científicas, Ramiro de Maeztu, Madrid, Spain; 2 Proteomics Facility, Centro de Investigaciones Biológicas del Consejo Superior de Investigaciones Científicas, Ramiro de Maeztu, Madrid, Spain; 3 Section of Microbiology, Imperial College London, Flowers Building, Armstrong Road, London, United Kingdom; Dartmouth College, UNITED STATES

## Abstract

Intracellular traffic in *Aspergillus nidulans* hyphae must cope with the challenges that the high rates of apical extension (1μm/min) and the long intracellular distances (>100 μm) impose. Understanding the ways in which the hyphal tip cell coordinates traffic to meet these challenges is of basic importance, but is also of considerable applied interest, as fungal invasiveness of animals and plants depends critically upon maintaining these high rates of growth. Rapid apical extension requires localization of cell-wall-modifying enzymes to hyphal tips. By combining genetic blocks in different trafficking steps with multidimensional epifluorescence microscopy and quantitative image analyses we demonstrate that polarization of the essential chitin-synthase ChsB occurs by indirect endocytic recycling, involving delivery/exocytosis to apices followed by internalization by the sub-apical endocytic collar of actin patches and subsequent trafficking to TGN cisternae, where it accumulates for ~1 min before being re-delivered to the apex by a RAB11/TRAPPII-dependent pathway. Accordingly, ChsB is stranded at the TGN by Sec7 inactivation but re-polarizes to the apical dome if the block is bypassed by a mutation in *geaA*^*gea1*^ that restores growth in the absence of Sec7. That polarization is independent of RAB5, that ChsB predominates at apex-proximal cisternae, and that upon dynein impairment ChsB is stalled at the tips in an aggregated endosome indicate that endocytosed ChsB traffics to the TGN *via* sorting endosomes functionally located upstream of the RAB5 domain and that this step requires dynein-mediated basipetal transport. It also requires RAB6 and its effector GARP (Vps51/Vps52/Vps53/Vps54), whose composition we determined by MS/MS following affinity chromatography purification. Ablation of any GARP component diverts ChsB to vacuoles and impairs growth and morphology markedly, emphasizing the important physiological role played by this pathway that, we propose, is central to the hyphal mode of growth.

## Author summary

Filamentous fungi form long tubular cells, called hyphae, which grow rapidly by apical extension, enabling these sessile organisms to explore substrates and facilitating tissue invasion in the case of pathogenic species. Because the shape of the hyphae is determined by an external cell wall, hyphal growth requires that cell-wall sculpting enzymes polarize to the tips. Endocytosis is essential for hyphal growth, and it was suspected that this results from its participation in a recycling pathway that takes up cell-wall enzymes from the plasma membrane and re-delivers them to the apex. Here we track the trafficking of a chitin synthase (a cell-wall modifying enzyme) to demonstrate that it is polarized by endocytic recycling. This chitin synthase is delivered by exocytosis to the apex, but diffuses away until being captured by a subapical collar of actin patches (sites of endocytosis) from where it reaches a sorting endosome before undergoing transport to the nearest trans-Golgi cisternae and incorporating into secretory vesicles that re-deliver the enzyme to the apex. Because impairing transit across this pathway compromises apical extension markedly and results in severe morphological defects, the pathway could be manipulated to prevent fungal pathogenicity of plants and humans, an enormous burden on human welfare.

## Introduction

Fungal pathogenicity of plants and animals constitutes an enormous burden on human welfare (reviewed by [1]), bequeathing a compelling case for understanding basic fungal biology. A characteristic feature of filamentous fungi is a vegetative phase consisting of tubular cells—hyphae—that grow exclusively by apical extension. In the ascomycete *A*. *nidulans* hyphae arise from dispersal mitospores denoted conidiospores that, upon germination, establish a polarity axis that, if undisturbed, can relentlessly support growth by apical extension. Thus polarized growth is a distinctive feature of hyphal fungi, and one that underlies their capacity to colonize substrates or, in the case of pathogenic species, invade live tissue.

Hyphal shape is determined by a cell wall that is formed by *de novo* synthesis as hyphal tip growth proceeds [2]. Thus, to sustain the strikingly rapid rates of growth (circa 1 μm/min at 30°C in *A*. *nidulans*) [3] the secretory pathway must efficiently deliver to the apex the enzymes that synthesize the cell wall in the hyphal tip dome and the lipids required for the increase in plasma membrane (PM) surface. Many fungi streamline this delivery by gathering a stock of secretory vesicles (SVs) at a structure denoted the Spitzenkörper (SPK), adjacent to the apical PM. According to the widely accepted model of hyphal growth [4–9], the SPK acts as a vesicle supply center that stores SVs before they are tethered to, and fuse with, the plasma membrane (PM) at the apical surface [4–9]. Vesicles at the SPK are loaded with cell wall-modifying enzymes (CWMEs) [10–14], demonstrating that these cargoes are exocytosed in a polarized fashion. Yet considering that key CWMEs such as β(1–3) glucan synthase [12] and chitin synthases [15] are integral membrane proteins, apical delivery alone cannot account for their polarization, which additionally requires a mechanism(s) that counteracts retrograde diffusion across the PM.

Work in *S*. *cerevisiae* demonstrated that localized exocytosis coupled to the rapid endocytic recycling of the synaptobrevin homologue Snc1p generates polarity [16]. The concentration of the endocytic internalization machinery of *A*. *nidulans* and many other fungi in a collar behind the hyphal tip [17–19], conveniently located to serve as diffusion barrier for integral membrane proteins, led us and others to suggest that the *A*. *nidulans* synaptobrevin SynA [18,20–22] and the phospholipid flippase DnfA [14] are polarized in the apical dome by endocytic cycling. This kinetic polarization mechanism implies that cargo taken up by endocytosis must return rapidly to the PM by exocytosis, as indirectly supported by studies with FM4-64 [23]. However, the identity of the compartment(s) involved in these recycling pathways(s) has not been characterized, nor has their physiological role been established, even though they represent the quintessence of the fungal lifestyle.

The different compartments of the *A*. *nidulans* secretory pathway (ER [24,25], early Golgi (EG) [26,27] and trans-Golgi network (TGN) [28]) have been characterized. The TGN delivers clathrin-coated carriers to the endosomal system [29] and SVs to the SPK [6]. The biogenesis of these SVs at TGN cisternae involves the recruitment of RAB11 mediated by the TRAPPII oligomeric GEF complex [6,30] and the subsequent engagement of kinesin-1 and myosin-5 motors that cooperate to transport SVs to the SPK [3,31], from which they reach the apical PM. [3,31]

Here we track the endocytic recycling pathway that determines the apical delivery and tip localization of CWMEs using fluorescent tagged versions of ChsB, a chitin synthase specific to filamentous fungi that is crucial for hyphal growth [32,33]. We demonstrate that ChsB is polarized to the apical dome by indirect endocytic recycling, such that the enzyme that diffuses away from the apex is internalized by the subapical collar of actin patches, re-routed from endosomes to Sec7-containing TGN cisternae in a GARP- and Rab6-dependent manner and subsequently re-delivered to the apex.

## Results

### The nearly essential chitin synthase ChsB appears to be a cargo of endocytic recycling

**Fig 1:**
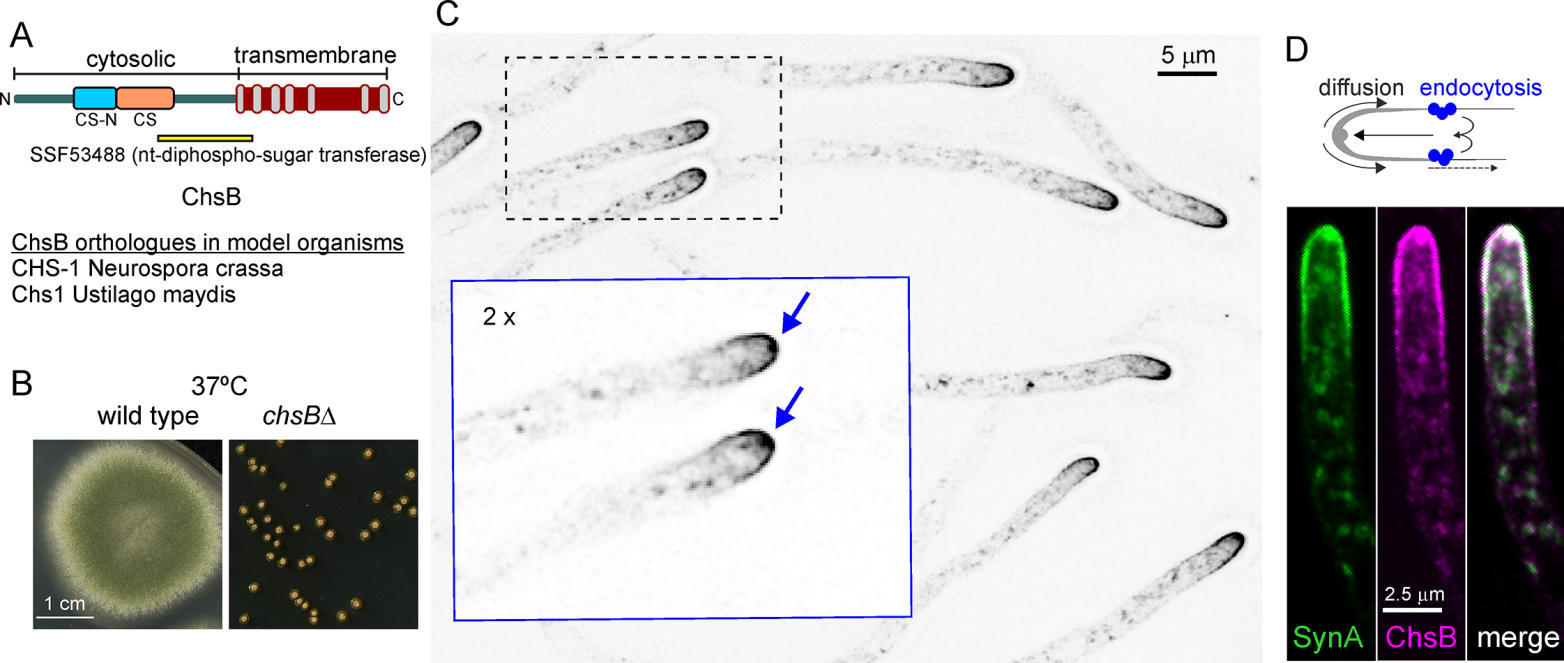
Polarization of ChsB. (A) domain organization of ChsB. CS-N, Chitin_synth_1N (PF08407); CS, Chitin_synth_1 (PF01644); the C-terminal transmembrane region includes 7 predicted helices (gray boxes). (B) Growth phenotype of *chsBΔ* microcolonies compared to the wt; plates incubated for 3 days at 37°C. (C) Subcellular localization of endogenously tagged GFP-ChsB. Arrows in the magnified inset indicate the Spitzenkörper (SPK). The image is a MIP of a deconvolved z-stack. (D) The synaptobrevin homologue SynA and ChsB strictly colocalize in the apical crescent, besides the SPK. Images are MIPs of deconvolved z-stacks. The scheme shows an interpretation of endocytic recycling.

ChsB is a chitin synthase belonging to class III (a class without representatives in yeasts) [33,34]. It is an integral membrane protein consisting of a 543-residue cytosolic domain and a 372-residue heptahelical membrane domain (Fig 1A). We chose this protein as model cargo to track endocytic recycling for two reasons: firstly, it has been determined that ChsB is polarized [32,35], suggesting that this enzyme is a cargo of endocytic recycling; secondly, *chsBΔ* is virtually lethal [32,33], resulting in microcolonies that do not progress (Fig 1B). That ChsB is physiologically crucial implies that altering its subcellular localization is directly translated into growth reductions/abnormal cell morphology. Thus, to determine if fusions of ChsB with fluorescent proteins are functional, we tagged the gene endogenously, which additionally minimized the possibility of perturbing the trafficking/steady state localization of ChsB with overexpression. C-terminal tagging was very debilitating, whereas N-terminal tagging did not affect growth (S1 Fig), indicating that the N-terminal fusion proteins fulfill the physiological role of ChsB. GFP-ChsB and mCherry-ChsB were indistinguishable from each other in localization (S1 Fig).

ChsB localizes to the SPK and to the PM of the tip, extending 3–4 μm behind the apex in rapidly growing hyphae (Fig 1C). We will refer to this PM region as ‘the apical dome’. In addition, ChsB localizes to internal puncta resembling Golgi cisternae. The localization of ChsB to the apical dome suggested that once the enzyme is delivered to the PM by secretory vesicles (SVs) derived from the SPK, it diffuses away from the apex. However, its diffusion is restricted to the tip region because ChsB is efficiently taken up by endocytosis at the subapical collar of actin patches (Fig 1D scheme). Indeed when we combined mCherry-ChsB with GFP-SynA, a v-SNARE undergoing endocytic recycling [18,20], both proteins displayed identical localization (Fig 1D), indicating that they use similar mechanisms to polarize.

### The subapical endocytic ring takes up ChsB

**Fig 2:**
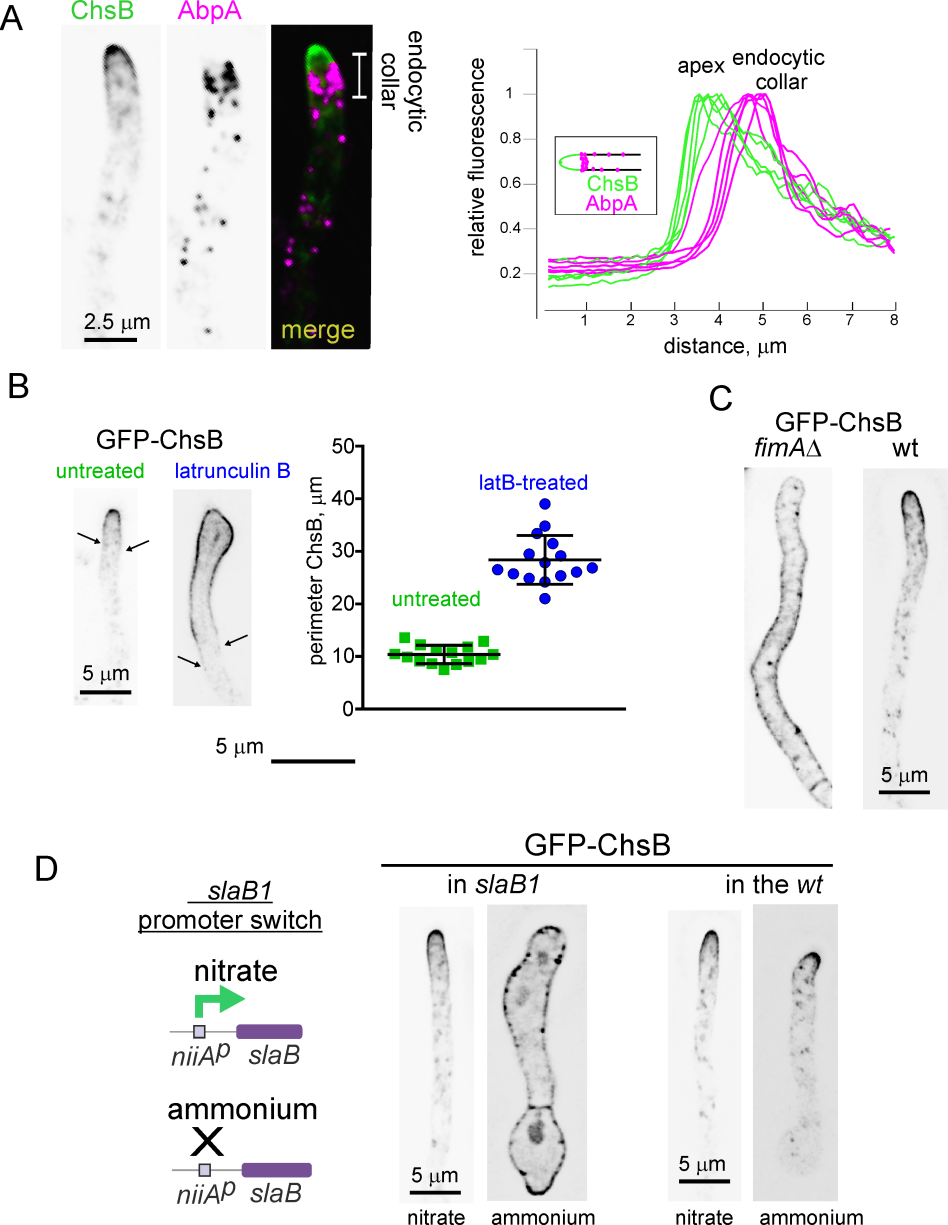
Endocytosis required to maintain ChsB polarity. (A) The basal limit of the GFP-ChsB apical crescent coincides with the position of the endocytic collar labeled with mCh-AbpA (actin binding protein1). Right graph, linescans along the longitudinal hyphal axis for the green (ChsB) and red (AbpA) channels. (B) Latrunculin B (100 μM) treatment facilitates the diffusion of GFP-ChsB to plasma membrane regions located far away from the apex. Arrows indicate the limits of the PM region occupied by ChsB in untreated and treated examples. The perimeters of the regions occupied by GFP-ChsB in the PM of treated and untreated hyphae are plotted on the right (n = 15 tips). Error bars indicate S.D. The two populations, which passed normality tests, are significantly different (P < 0.0001) in an unpaired *t*-test with Welch’s correction. (C) Localization of ChsB in a *fimAΔ* hypha compared to the wt. (D) Scheme: *slaB1* drives expression of SlaB under the control of the nitrite reductase promoter (*niiA*^*p*^); Images show the localization of ChsB in a strain carrying the conditional expression allele *slaB1* as the only source of SlaB^Sla2^ and its comparison with the wt. *slaB1* drives expression of this key endocytic regulator on nitrate as N source but not on ammonium. The germlings derived from conidiospores continuously cultured on medium containing nitrate or ammonium, as indicated. All images represent MIPs of deconvolved z-stacks.

We used several approaches to demonstrate that the endocytic collar indeed takes up ChsB. Firstly we imaged GFP-ChsB with the endocytic patch marker AbpA-mRFP [17], which showed that the distal limit of the ChsB apical dome coincides with the localization of the endocytic collar (Fig 2A). S1 Movie illustrates the spatial coupling of the endocytic collar and the ChsB apical dome, which move forward concertedly as hyphal tip growth proceeds, as demonstrated for SynA [18]. Secondly we added the F-actin depolymerizing drug latrunculin B (latB) to growing hyphae, which disassembles endocytic actin patches [28]. This treatment resulted in ChsB outspreading beyond the tip region (Fig 2B)(the average perimeters ± S.D. of the PM region occupied by ChsB were 10.4 ± 1.7 μm and 28.4 μm ± 4.6 in untreated and treated hyphae, respectively). As exocytosis is largely prevented by actin depolymerization and as secondary polarity axes were not observed, this finding implies that the spreading of ChsB results from unrestricted diffusion of the enzyme previously delivered at the apex by exocytosis, strongly implicating endocytosis in confining ChsB localization to the apical dome.

Thirdly we determined the localization of ChsB in a null *fimAΔ* mutant, which is severely impaired in endocytosis [19]. *fimAΔ* results in morphogenesis defects, yet a proportion of the cells gave rise to hyphae, and these hyphae showed uniform distribution of ChsB in the PM (Fig 2C). The fact that ChsB localizes to the plasma membrane of the mutant implies that *fimAΔ* does not block ChsB exocytosis, which is consistent with previous work demonstrating that the seven transmembrane domain pH signaling receptor PalH localizes to the PM in a *fimAΔ* background, showing that *fimAΔ* does not prevent the delivery of a polytopic exocytic protein to the PM either [22]. Thus the uniform distribution of ChsB in *fimAΔ* hyphae appears to result from unrestricted diffusion of ChsB resulting from the endocytic defect.

To corroborate this conclusion we used the conditional expression allele *slaB1* [36], based on the nitrate reductase promoter, allowing the synthesis of the key endocytic regulator SlaB in cells cultured on nitrate as nitrogen source but not on ammonium. The absence of SlaB virtually blocks endocytosis, allowing cells to establish polarity but not to maintain it efficiently, such that conidia germinated on ammonium give rise to a population of morphologically abnormal germlings containing short hyphal tubes [36]. These *slaB1* germlings accumulate ChsB throughout their entire PM (Fig 2D), unlike wt cells of a similar length, which contain polarized ChsB. This uniform and predominant localization of ChsB to the PM in the *slaB1* background closely resembles the localization of the also exocytic cargo PalH [22], and could not be explained if *slaB1* prevented exocytosis.

**Fig 3:**
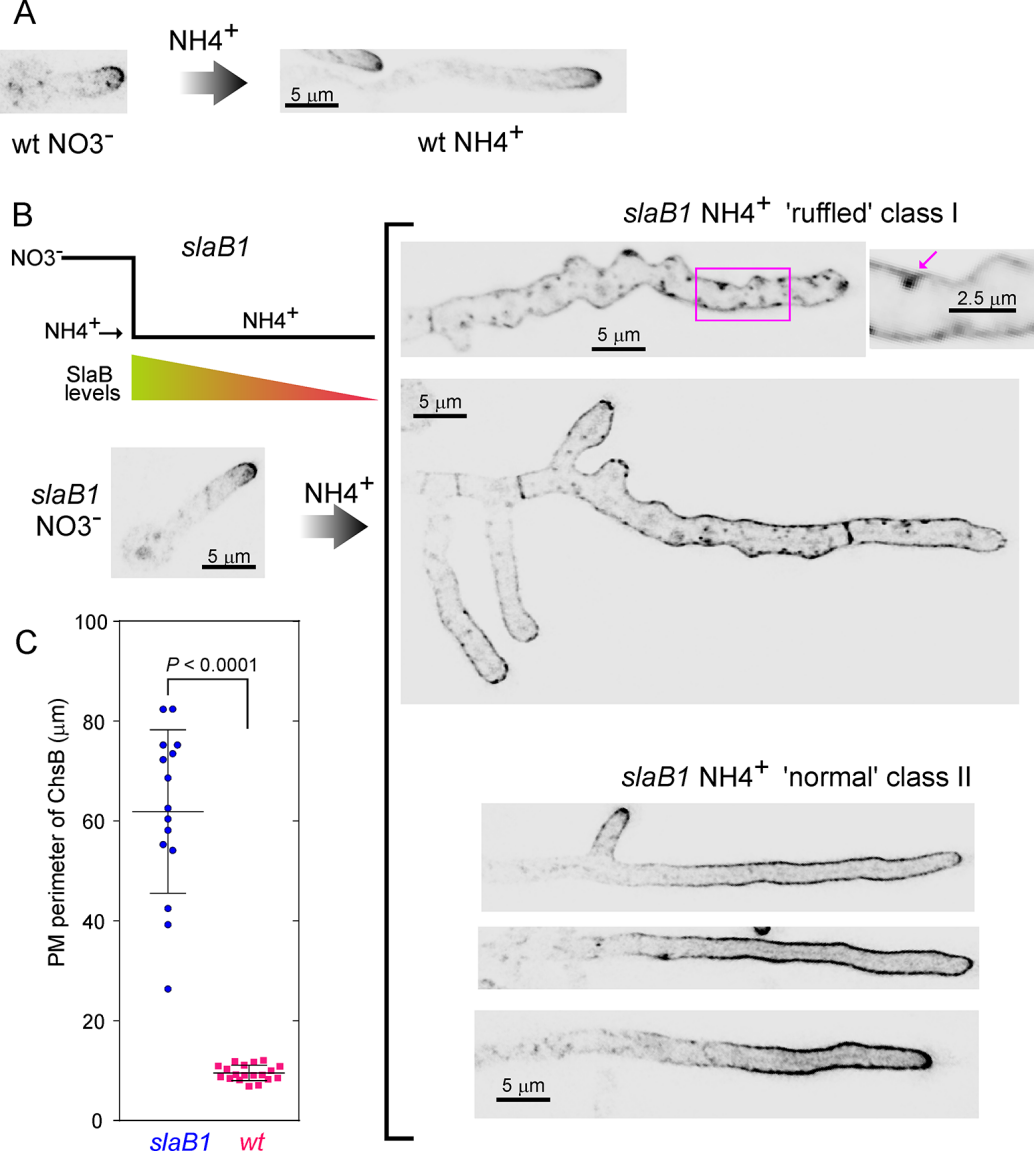
SlaB downregulation results in ChsB depolarization. (A) GFP-ChsB localization in hyphae derived from germlings that had been pre-cultured on medium containing nitrate as sole N source (left) and subsequently shifted to medium containing ammonium (right). (B) Promoter down-shift experiment with *slaB1*. The same nutritional regime used in (A) results in downregulation of SlaB levels (scheme), markedly affecting the polarization of GFP-ChsB in the PM. Class I (‘ruffled’) and class II (‘normal’) hyphae are depicted. For class I the inset shows a characteristic GFP-ChsB ‘clump’ associated with the PM (arrowed). (C) Quantitation of the perimeter of PM occupied by ChsB in wt (n = 19) and *slaB1* (n = 15) cells pre-cultured on nitrate and shifted to ammonium. The two datasets were significantly different (*P* < 0.0001) in an unpaired *t*-test. All images represent MIPs of deconvolved z-stacks and are shown at the same magnification, with the exception of the inset, which is magnified 2.5 times.

Next, to show that ChsB diffusion resulting from an endocytic deficit occurs from the tips, we used *slaB1* in a promoter down-shift experiment. To this end we pre-cultured wt and *slaB1* germlings on nitrate (Fig 3A and 3B, left) and shifted them to ammonium. The shift had no effect on ChsB localization to the apical dome in the wt (Fig 3A, right). In contrast, it progressively depleted SlaB^Sla2^ in *slaB1* hyphae, which continued growing for several hours using the protein synthesized before the promoter was shut off (Fig 3B, scheme). After this time, the resulting population of *slaB1* hyphae could be classified in two classes. One class consisted of cells with ‘ruffled’ appearance in which ChsB localizes, depolarized, at the PM, which often shows clumps of ChsB (Fig 3B, top right). In *slaB1* cells shifted to ammonium SynA also forms similar clumps, which have been shown to associate with invaginations of the PM resulting from endocytic block [36]. Thus this class of cells appears to correspond to hyphae in which endocytosis was essentially blocked. Their ‘ruffled’ appearance indicates the collapse of the main polarity axis, with concomitant activation of secondary polarity axes from which ChsB spreads away (Fig 3B, top right). The marked impairment of polarity maintenance that such profusion of secondary polarity axes reveals suggests that strong impairment of endocytosis might affect polarity landmarks/determinants (see discussion).

The second *slaB1* class consisted of morphologically normal hyphae, in which the major polarity axis had been maintained and that did not show clumps of ChsB in the PM (Fig 3B, bottom right), consistent with these cells reflecting a stage in which endocytosis is impaired, but to a lesser extent. Remarkably, in these cells ChsB spread away from the tips (compare with the wt in Fig 3A), such that the perimeter of PM occupied by the reporter was ~6 times greater than in wt controls (Fig 3C, average values were 62.5 ± 16.4 S.D. and 9.5 ± 1.5 S.D. in *slaB1* and wt cells, respectively). These results show that that downregulation of endocytosis permits unrestricted diffusion of ChsB delivered to the apical surface, resulting in its dispersion away from the apical dome. Therefore we conclude the polarization of ChsB to the apical dome requires endocytosis.

### Within the cell ChsB localizes to the tip-proximal cisternae of the TGN.

**Fig 4:**
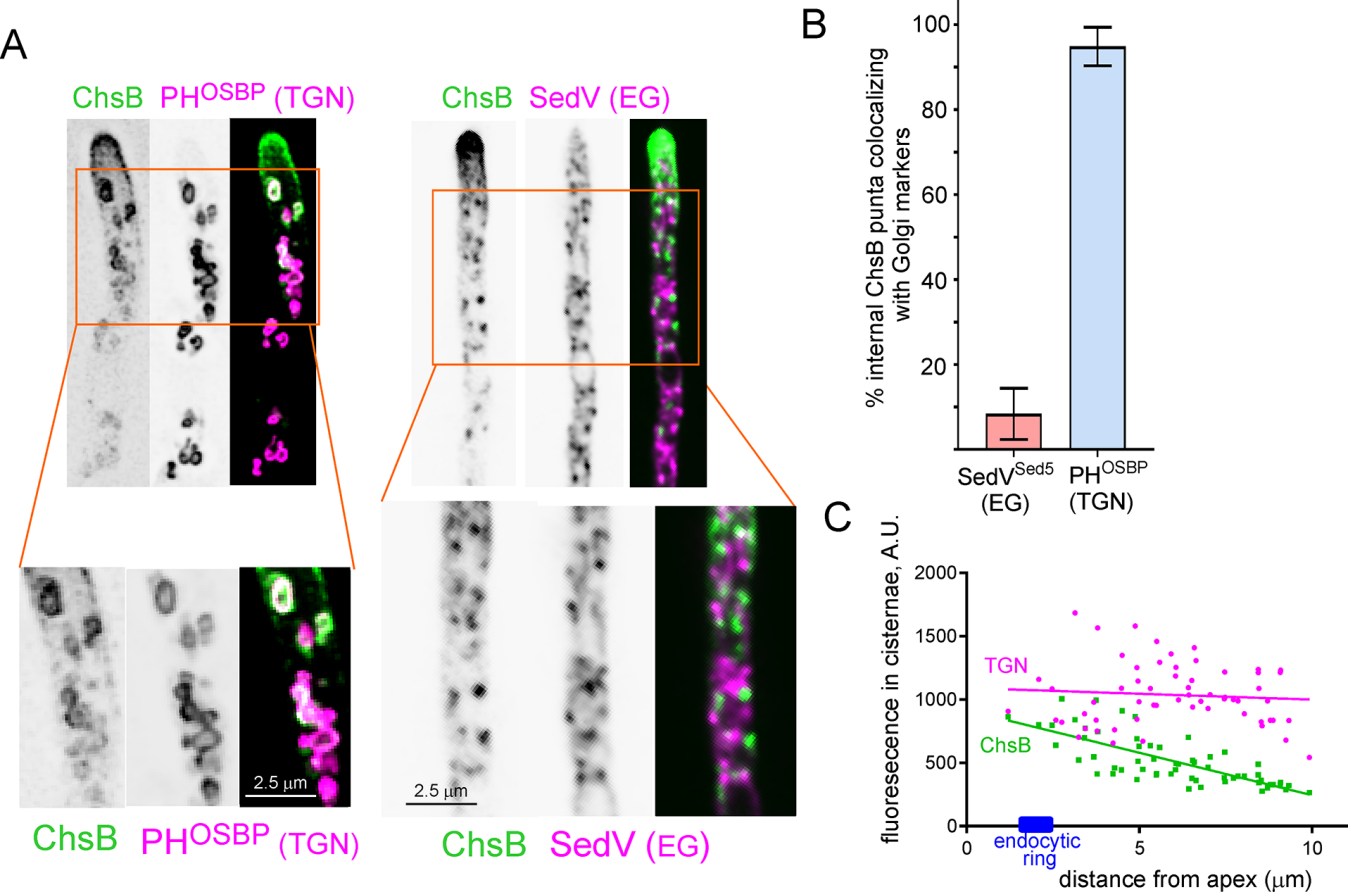
ChsB localizes to the tip-proximal cisternae of the TGN. (A) colocalization of internal puncta containing GFP-ChsB with the TGN marker mRFP-PH^OSBP^, and absence of colocalization with the early Golgi marker mCh-SedV^Sed5^ (syntaxin 5). Note the characteristically fenestrated structures of the TGN puncta in the left panels. (B) Quantitation of internal ChsB structures that contain Golgi markers for 178 puncta in n = 10 mRFP-PH^OSBP^ hyphae and 122 puncta in n = 11 mCh-SedV^Sed5^ hyphae. Error bars indicate mean ± SD. The two datasets were significantly different (*P*< 0.0001) in an unpaired t-test. (C) Plot of fluorescence intensities in the PH^OSBP^ and the ChsB channels vs. distance to the apex. Data of *n* = 60 TGN cisternae were pooled from 6 hyphae. The fluorescence of ChsB negatively correlates with the distance to the apex (Pearson’s *r* = -0.748, *P* = 6E-12) whereas that of PH^OSBP^ does not (*r* = -0.08, *P* = 0.53).

What is the internal compartment consisting of punctate structures where ChsB resides? These structures resembled Golgi cisternae [28], which in *A*. *nidulans* are not stacked, being therefore resolvable by optical microscopy [21]. Thus we investigated the Golgi localization of ChsB with the early Golgi SedV^Sed5^ syntaxin and with the TGN reporter PH^OSBP^; Fig 4A). ChsB and PH^OSBP^ showed almost complete colocalization: 95% of 178 ChsB puncta in *n =* 10 hyphae were labeled with PH^OSBP^, contrasting with only 8% of 122 ChsB puncta in *n =* 11 hyphae containing SedV^Sed5^ (Fig 4B). Thus ChsB localizes to the TGN. However, not every TGN cisternae contained similar levels of ChsB, with the highest intensity of ChsB signal corresponding to the apex-proximal ones (Fig 4A). To illustrate, we quantified ChsB fluorescence in 60 TGN cisternae located within the apical-most 10 μm of *n =* 6 hyphae and compared the resulting values with those for PH^OSBP^. Cisternal ChsB fluorescence negatively correlated with the distance to the apex (*P* = 6E-12, *r* = -0.75, *n* = 60), whereas PH^OSBP^ fluorescence did not (Fig 4C). Thus, this key observation suggested that cisternae that are closer to the endocytic collar accumulate more ChsB, arguably because they would be first to receive ChsB taken up by endocytosis. It further suggested that recycling of ChsB proceeds, at least in part, by way of the TGN, rather than directly from a sorting endosome. This is an important conclusion, as a direct pathway connecting sorting endosomes with the PM has been recently described in the related ascomycete *S*. *cerevisiae* [37]

**Fig 5:**
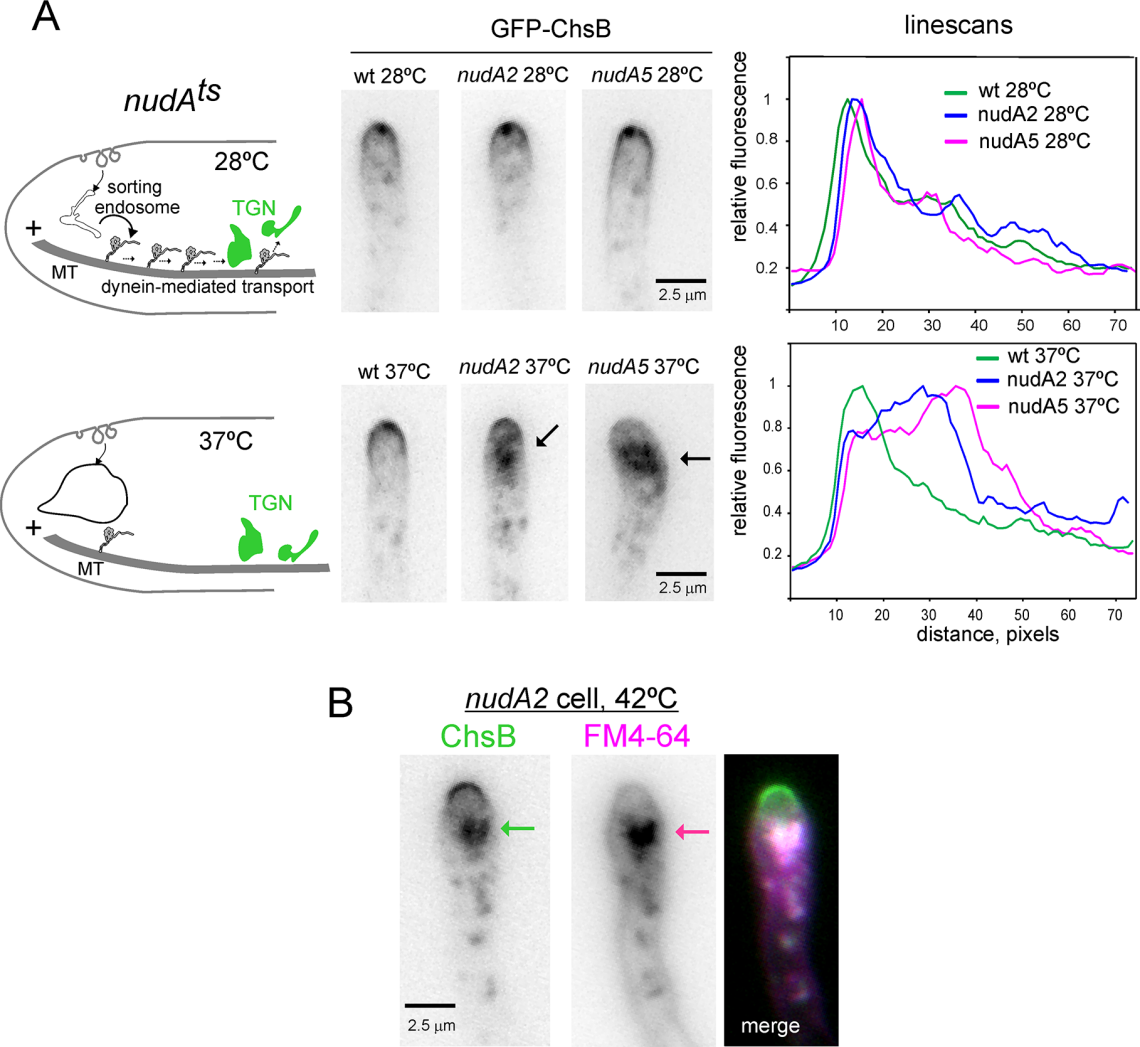
**Recycling of ChsB from endosomes necessitates dynein** (A) Left, schematics summarizing the rationale of these experiments. Middle images: localization of GFP-ChsB in hyphal tips of the wt and of strains carrying ts mutations in *nudA* encoding the dynein heavy chain, before and after shifting cells from 28°C to 37°C. Note the aggregate of ChsB in the *nudA* mutants at 37°C (see scheme), which forms apparently at the expense of the signal in the apical dome and the SPK (which is not detectable in the mutants at 37°C). Right, linescans of the ChsB channel for the hyphae displayed in the images (1 px, 0.103 μm). (B) Hyphal tip of a *nudA2* cell shifted to 42°C, stained with the endocytosed membrane tracker FM4-64.

The greater abundance of ChsB in the apicalmost TGN cisternae additionally suggested that retrograde (here meaning ‘away-from-the-apex’) transport connects the endosomal compartments that receive traffic from the subapical collar of actin patches with the TGN. If so, the plus-end-towards-the-apex polarity of microtubules at the tips implies that such transport might involve dynein. We investigated this possibility with two *ts* mutations, *nudA2* and *nudA5*, affecting the dynein heavy chain, which behave as hypomorphs at 37°C [3,38]. ChsB distribution was normal in *nudA2* and *nudA5* cells at 28°C. However, when these cells were shifted to 37°C for ~2 h (which did not affect ChsB localization in the wt), ChsB delocalized to an intracellular aggregate in the tip region. The phenotype was very homogeneous within the mutant populations (to illustrate we determined that, for *nudA5*, 94% of *n* = 68 tips contained the tip aggregate, contrasting with none of *n* = 74 wt tips). The abnormal ChsB aggregate of the *nudA* mutants formed apparently at the expense of the PM/SPK pool, which was markedly diminished (Fig 5A). Of note, under similar conditions *nudA2* and *nudA*5 do not prevent the accumulation of RabE^RAB11^ SVs at the SPK [3] indicating that the ChsB aggregate is not exocytic. Indeed we determined that this *nud* cell compartment in which ChsB stalls is readily accessible (5 min after dye loading) to the endocytic tracer FM4-64 [39], confirming that it has early endocytic origin (Fig 5B). In contrast this compartment does not stain with the late endosome tracer CMAC [20]. Thus the above results strongly indicate that traffic between endosome compartments at which endocytosed ChsB initially arrives and the TGN involves dynein.

### ChsB resides at the TGN only transiently

**Fig 6:**
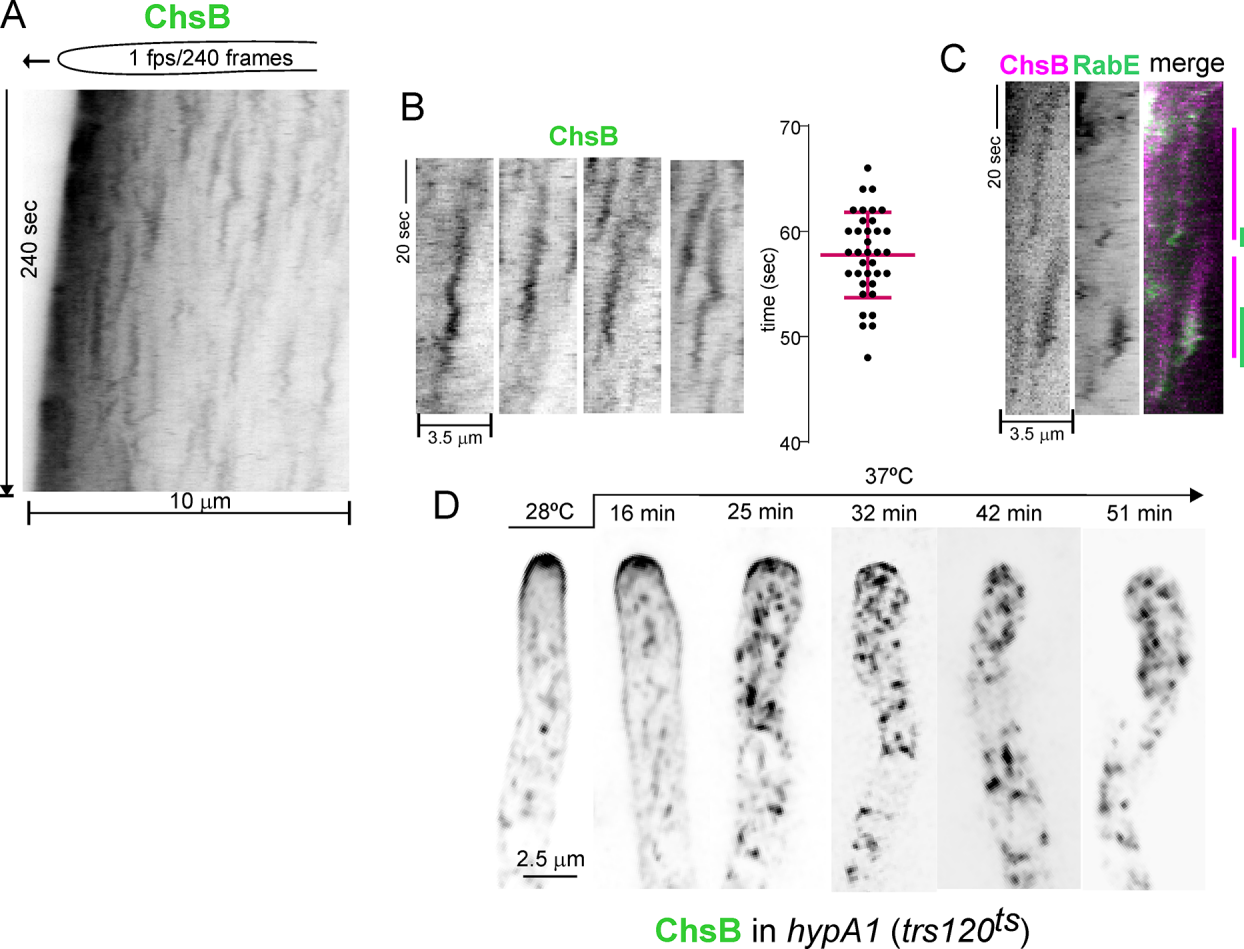
Dynamics of ChsB in the TGN. (A) GFP-ChsB is a transient resident of TGN cisternae: kymograph traced along the longitudinal axis of a growing hypha showing multiple events of transient ChsB recruitment to TGN cisterna. The strong signal at the apex is the SPK. (B) Selected examples of events of ChsB recruitment to TGN cisternae and statistical analysis of the average residence time of ChsB on them (58 ± 4 S.D., *n* = 36); (C) region of a kymograph showing two examples of RabE^RAB11^ being recruited at the end of the ‘ChsB cycle’. The green and red channels of a cell co-expressing mCh-ChsB and GFP-RabE^RAB11^ were filmed simultaneously with a beam splitter at 1 fps time resolution (D) *hypA1*^*ts*^ cells (*hypA* encodes *A*. *nidulans* Trs120 in TRAPPII) expressing GFP-ChsB were filmed at 28°C and at different times after shifting the culture to 37°C on the microscope stage. The time required for the culture medium to reach 37°C is ~15 min. Note the delocalization of ChsB at the apical dome and the SPK to internal structures.

Next we determined the fate of ChsB in the TGN. Time-lapse microscopy (time resolution 1–2 fps) showed that TGN cisternae acquire ChsB only transiently. Fig 6A is an example kymograph representing the intensities of the cytosolic ChsB puncta *vs*. time (in the *y* axis). In this plot the ‘lengths’ of the ‘vertical’ lines’ slightly tilted in the direction of growth reflect the residence time of ChsB in the TGN cisternae. The average residence time, determined with *n =* 36 events, was 58 ± 4 sec S.D. (Fig 6B), which is about half the 2 min average lifetime previously determined for TGN cisternae [6]. This suggested that the duration of ChsB at the TGN would be bound by its arrival to an existing cisterna and its departure from it, incorporated as cargo of SVs that are delivered to the SPK before undergoing fusion with the PM. ChsB and RabE^RAB11^ strictly colocalize at the SPK over time (S2 Movie), implicating RabE^RAB11^ carriers in ChsB transport between the TGN and this structure. Because the background ‘noise’ due to the strong predominance of ChsB in the tip region impeded tracking of ChsB-containing SVs on their way to the SPK we attempted to investigate the exiting of ChsB indirectly, by correlating the end of the ChsB cycle with the arrival of RabE^RAB11^ to cisternae. We detected a few isolated events in which GFP-RabE^RAB11^ was recruited at the end of the mCh-ChsB cycle (Fig 6C), but these experiments were hampered by photobleaching. Thus, to determine the involvement of RabE^RAB11^ we asked if disabling the RabE^RAB11^ GEF TRAPPII [30] impeded ChsB localization to the SPK and the PM using the *hypA*1 mutation that inactivates Trs120, a key component of the TRAPPII complex. Fig 6D shows that following a shift to 37°C *hypA*1 cells delocalized ChsB, which gradually disappeared from the SPK and the apical dome to internal structures. Thus ChsB resides at the TGN only transiently and RabE^RAB11^ is necessary for the delivery of ChsB to the SPK and the PM.

### ChsB is stranded in the TGN if Sec7 is inactivated

**Fig 7:**
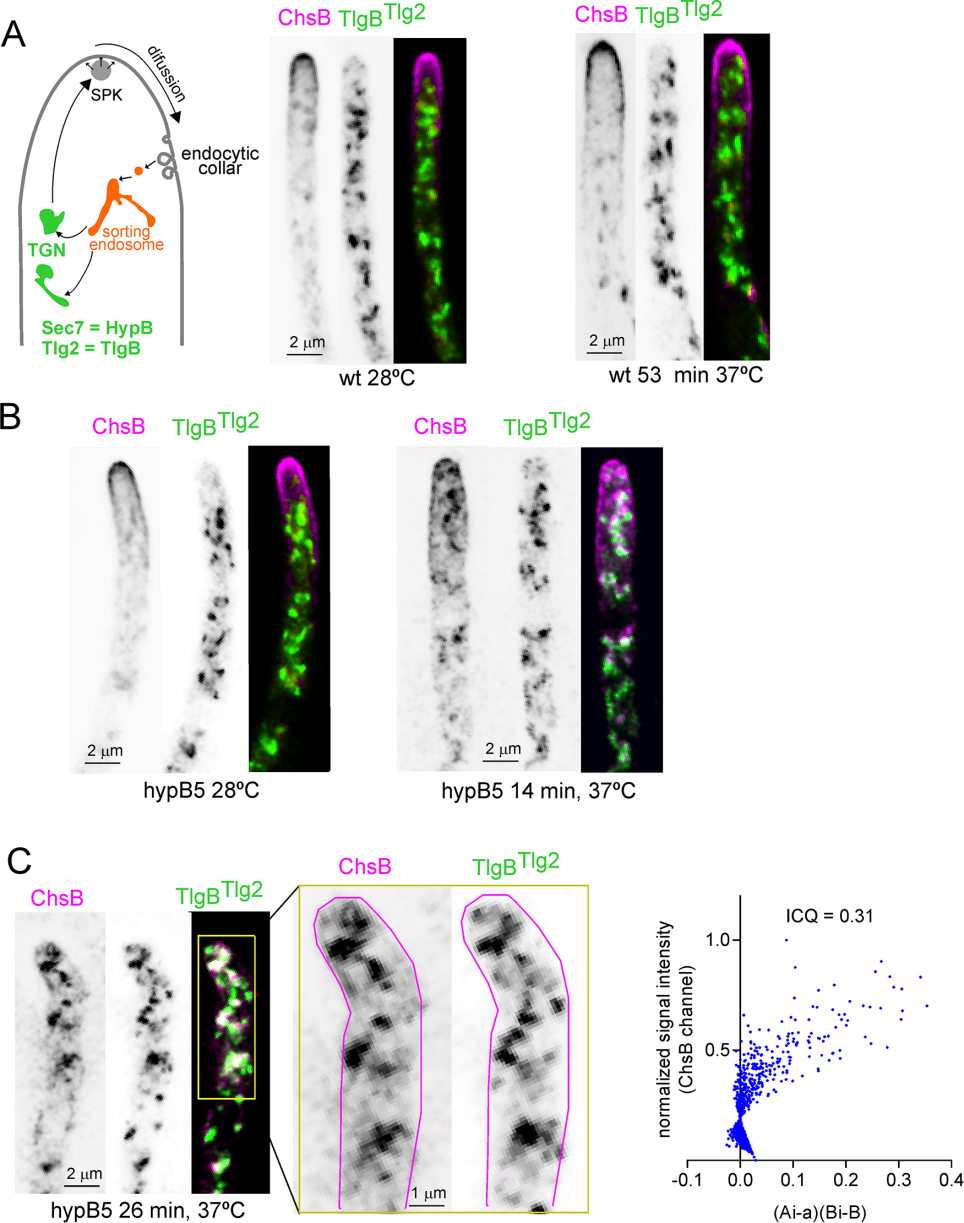
A *hypB5* (= *sec7*^*ts*^) mutation strands ChsB in the TGN. (A) Images of a wt strain co-expressing mCh-ChsB and GFP-TlgB cultured at 28°C and following a shift to 37°C. (B) Images of the corresponding *hypB5* strain before and 14 min after the temperature shift. (C) The *hypB5* mutant at a later time-point. Right graph, plot of Li’s intensity correlation coefficient (ICQ) used to estimate colocalization. ‘Ai’ and ‘a’ are the ChsB channel’s current and mean intensity, whereas ‘Bi’ and ‘b’ indicate the same values for the TlgB^Tlg2^ channel. Colocalization results in a pixel cloud spread on the right side of the plot. ICQ ranges from −0.5 (exclusion) to 0.5 (complete colocalization). All images are MIPs of deconvolved z-stacks.

The *S*. *cerevisiae* SynA orthologue Snc1 traffics from endosomes to the TGN before returning to the PM [40]. However, the existence of a second pathway mediating recycling directly from sorting endosomes has been demonstrated recently in *S*. *cerevisiae* [37]. A diagnostic feature of this ‘direct’ pathway is that its cargo does not accumulate in TGN cisternae in non-permissive conditional *sec7*^*ts*^ cells [Sec7 is the ARF1 GEF at the TGN [41]]. As our data above strongly suggested that ChsB undergoes endocytic recycling indirectly, by way of the TGN (Fig 7A scheme), we imposed a genetic block using *hypB5*, a *ts* mutation in the gene encoding *Aspergillus* Sec7. To simultaneously monitor the effects of *hypB5* on ChsB localization and on the organization of the Golgi, we used a mCh-ChsB strain co-expressing the GFP-tagged t-SNARE TlgB^Tlg2^ [42] to track TGN cisternae with an integral membrane protein. Neither ChsB nor TGN cisternae were affected by *hypB5* at 28°C (Fig 7B), nor did a temperature shift to 37°C affect them in the wt (Fig 7A). In sharp contrast, in *hypB5* cells ChsB largely relocalized from the SPK and PM to internal structures at ~15 min after the shift. These ChsB-containing structures were closely associated (Fig 7B), and to some extent colocalized, even at this early time point, with the network of TlgB^Tlg2^ cisternae (Pearson’s coefficient = 0.47; Li’s intensity correlation quotient (ICQ) = 0.218)(S2 Fig). Between 15 and 30 min at 37°C internal ChsB structures and TlgB^Tlg2^ cisternae tended to aggregate, and colocalization became apparent (Fig 7C; Pearson’s = 0.74; Li’s ICQ = 0.31)(S2 Fig shows an extended set of examples; as reference, the two colocalizing TGN markers TlgB^Tlg2^ and PH^OSBP^ show and ICQ of 0.339, [26], see also Fig 7 legend). At ~40 min *hypB5* TGN cisternae aggregated but ChsB and TlgB^Tlg2^ still showed colocalization (S2 Fig). Thus ChsB accumulates in membranes with TGN identity after imposing a *sec7* block. Taken together, all the above data strongly indicated that the ChsB steady state localization at the SPK and the apical dome is maintained by endocytic recycling through the Sec7-containing TGN.

### A point mutation in GeaA^Gea1^ bypassing Sec7 restores ChsB localization to the apical dome

The *geaA1* mutation results in a Y1022C substitution in a conserved tripeptide of GeaA^Gea1^, the only early Golgi GEF of ARF1 [27]. Whereas wt GeaA localizes to the early Golgi, GeaA^Y1022C^ is slightly shifted towards the TGN and reaches the PM [27], suggesting that the mutated motif mediates the retention of GeaA in Golgi compartments. Remarkably *geaA1* substantially bypasses the essential role of Sec7 in the TGN [27].

**Fig 8:**
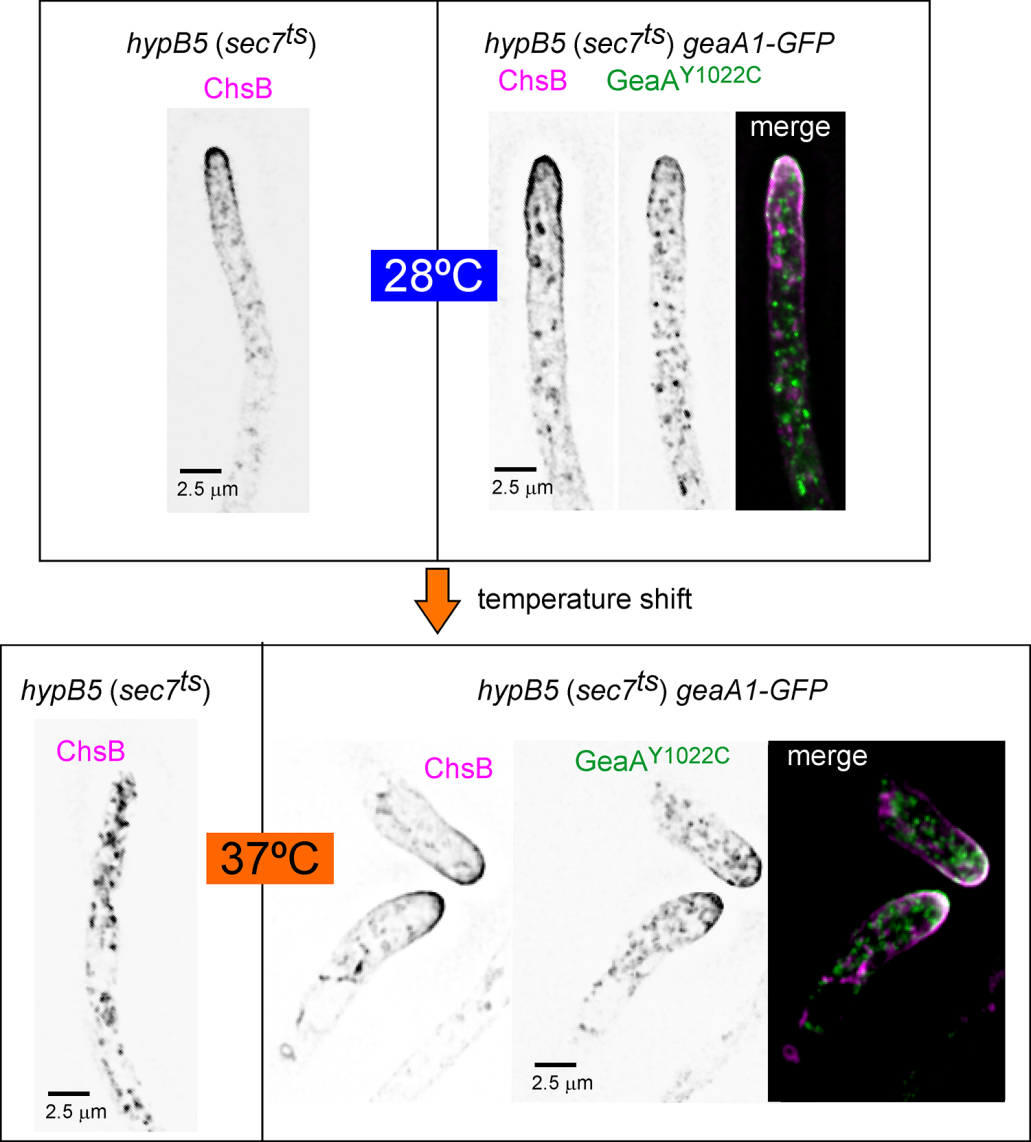
ChsB, stranded at the TGN by *hypB5* (= *sec7*^*ts*^), is rescued by *geaA1*. Images of *hypB5* mCh-ChsB strains carrying or not *geaA1-GFP* (encoding GFP-tagged GeaA^Y1022C^), photographed at 28°C or after a shift to 37°C for 27 min (*hypB5*), or 37 min (*hypB5* GFP-GeaA^Y1022C^). Note that GeaA^Y1022C^-GFP was the only source of GeaA^Gea1^ in the double mutant. Also note the colocalization of GeaA^Y1022C^ with ChsB in the apical dome at both temperatures.

To determine if *geaA1* suppresses the mislocalization of ChsB caused by *hypB5* we used an endogenously tagged *geaA1* allele encoding GFP-GeaA^Y1022C^, which suppresses *hypB5* as efficiently as the untagged allele [27]. GFP-GeaA^Y1022C^ enabled us to simultaneously follow the fate of both mCh-ChsB and the mutant ARF1 GEF in the *hypB5* (*sec7*^*ts*^) background. Fig 8A shows that at 28°C *geaA1-GFP* localized to an apical crescent and to internal Golgi structures as described [27], whereas ChsB localization was normal (i.e. to cytosolic puncta, the SPK and the apical dome). Next we tested whether *geaA1* rescued the *hypB5-*dependent delocalization defect of ChsB at 37°C. Indeed ChsB relocalized to the apical dome in *hypB5 geaA1* double mutant cells shifted to the restrictive temperature (Fig 8B, right), which contrasted markedly with the localization of ChsB to internal structures in *hypB5* single mutant controls under the same conditions (Fig 8B, left). Thus the polarized PM localization of ChsB necessitates Sec7 (HypB), and *geaA1* restoring the growth defect resulting from *hypB5* also restores the ChsB PM localization defects, directly implicating Sec7 in ChsB polarization.

### ChsB recycles to the TGN from an endosomal compartment located upstream of the RAB5 domain

**Fig 9:**
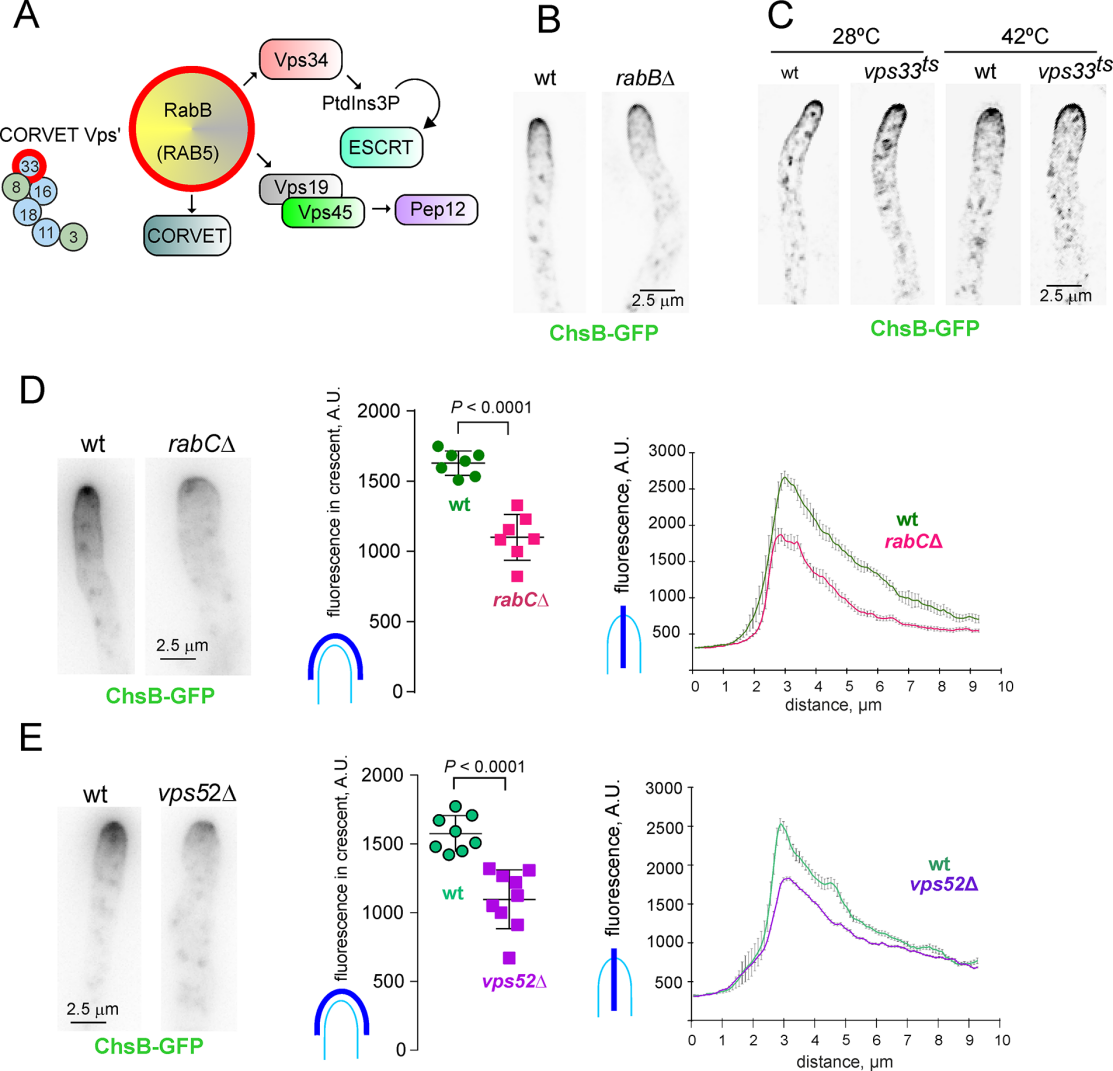
RabC^RAB6^- and GARP-dependent recycling of ChsB from an endosome located upstream of the RaB^RAB5^ domain. (A) Scheme of the effectors subordinated to RabB^RAB5^ in early endosomes (EEs)(see text). (B) Normal localization of ChsB in a *rabBΔ* mutant. (C) Normal localization of ChsB in a *vps33^ts^* strain at 28°C and following a shift to 42°C. (D) Left, delocalization of ChsB in *rabCΔ*; Middle, plot of average intensities of ChsB in wt and *rabCΔ* apical domes determined from 2 x 50 pixel arch-shaped linescans as in the scheme. *P* values estimated with an unpaired *t*-test; Right plot, linescans (mean values ± S.E.M. bars) of maximal intensities across the whole width of the same tips used for the plots. (E) Delocalization of ChsB from the apical dome in the *vps52Δ* mutant; middle and right plots and statistical analysis as in (D). Note that for (D) and (E), hyphal tip images were not deconvolved to better display the differences between the wt and the mutants.

RabB^RAB5^ is the major RAB acting in pre-vacuolar early endosomes (EEs) and a key driver of endosome maturation, as it recruits Vps34, the PtdIns3P-synthesizing kinase required for initiating the multivesicular body pathway [43]. It also recruits to endosomes the CORVET complex that mediates fusion events between endosomes as well as the microtubule-dependent motors facilitating their long-distant transport [20,43–47] (Fig 9A, scheme). Thus, if ChsB would recycle to the TGN from RAB5-containing endosomes a *rabBΔ* mutation should affect its localization strongly. However, ChsB was normal in a *rabBΔ* background (Fig 9B). Furthermore, *vps33-1*^*ts*^ affects the key SM protein Vps33 of the CORVET and HOPS complexes, thus playing an essential role in the maturation of endosomes [42,44,48,49]. However *vps33-1*^*ts*^ did not mislocalize ChsB at 42°C (Fig 9C), in agreement with *rabBΔ* data. Thus, the endosomal compartment from which ChsB recycles lies upstream of PtdIns3P-, CORVET- and ESCRT-containing EEs. This compartment, we hypothesize, is a loosely defined sorting endosome akin to the post-Golgi endosome proposed by Pelham and co-workers [50].

### ChsB recycling depends on GARP

To investigate the route that delivers ChsB from endosomes to the TGN we focused on RAB6 (RabC^RAB6^ in *Aspergillus*), a master regulator of retrograde traffic connecting endosomes with the Golgi [51]. A *rabCΔ* mutation [21] substantially diminished the amount of ChsB localizing to exocytic post-Golgi membranes, making it barely detectable at the SPK and substantially reducing its presence in the apical dome (Fig 9D). These data strongly implicated RabC^Rab6^ in ChsB recycling.

**Fig 10:**
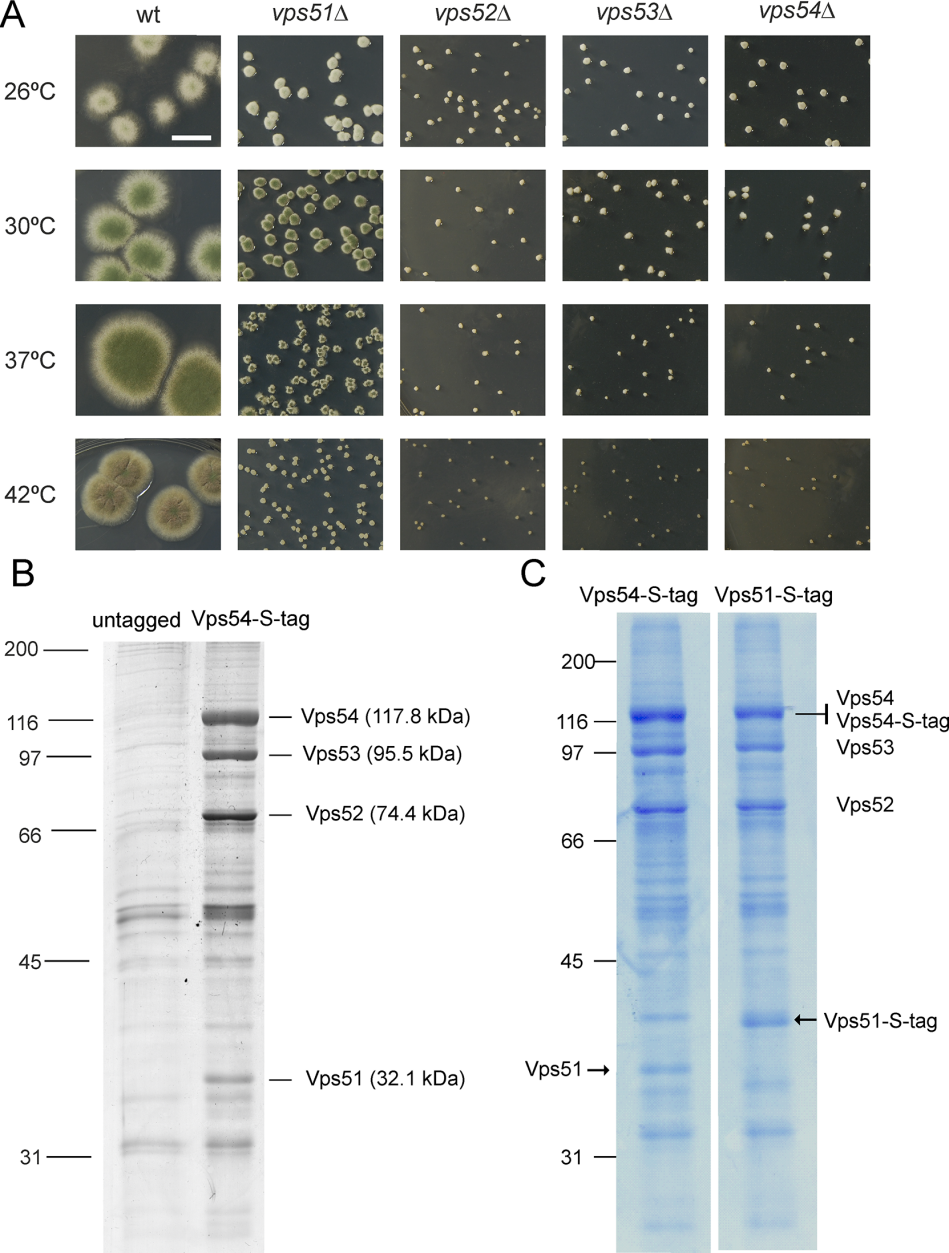
Characterization of *A*. *nidulans* GARP. (A) Growth of the wt and indicated mutant strains after 60 h of incubation. (B) Silver staining of proteins retained after passing extracts of a strain expressing endogenously tagged Vps54-S-tag compared with an untagged strain control. Proteins were eluted from S-tag columns. The indicated bands were excised and their identity determined by MS/MS. (C) Comparison of S-tag affinity purifications as in (B) but using Vps54-S-tag and Vps51-S-tag baits and colloidal Coomassie staining. Note the shift in mobility of Vps51 due to the S-tag.

A key effector of RAB6 proteins tethering fusion between endosome-derived retrograde transport carriers and the TGN is the Golgi-associated retrograde protein (GARP) complex, consisting of Vps51, Vps52, Vps53 and Vps54 in yeast [52–54]. We deleted the corresponding *Aspergillus* orthologues. Consistent with the corresponding gene products acting in a complex, all four deletion mutations were phenotypically similar in growth tests, markedly reducing colony size at all tested temperatures (Fig 10A), although *vps51Δ* was slightly less debilitating, as similarly reported in yeast [55]. Moreover, the fact that their colony phenotypes resembled that of *rabCΔ* [21] suggests that the growth-limiting function of RabC^Rab6^ involves GARP. *vps51Δ*, *vps52Δ vps53Δ* and *vps54Δ* further resembled *rabCΔ* in that all four mutations cause morphological aberrations and result in numerous small vacuoles (S3 Fig), consistent with the wt alleles playing the expected roles in endosome/Golgi interface [52].

The strong growth phenotype of *GARP* null mutations and the morphological effects that they cause indicated that GARP components are crucial for the organization of exocytic compartments. Indeed *GARP* mutations affected the TGN in two ways: (i) they caused depolarization of cisternae, which normally concentrate near the tip; (ii) they resulted in smaller cisternae lacking the characteristic fenestrated morphology of the wt (S4 Fig). These two phenotypes are shared with *rabCΔ* [21]. These observations are consistent with GARP capturing membranes delivered from endosomes to the TGN and further suggest that the normal polarization of TGN cisternae depends on this Rab6/GARP-mediated retrograde traffic.

To establish that *Aspergillus* Vps51, Vps52, Vps53 and Vps54 indeed associate to form GARP, we purified the complex from cell-free extracts using a single-step S-tag affinity purification protocol [56]. Firstly we pulled-down the complex with endogenously S-tagged (thus expressed at physiological levels) Vps54. Besides non-specific contaminants, silver staining of pulled-down material revealed four bands exclusive of Vps54-S-tag extracts. MS/MS spectrometry identified these bands as Vps54 (AN7993), Vps53 (AN2736), Vps52 (AN4014) and Vps51 (AN3015), but Vps51 appeared substoichiometric (Fig 10B). Thus we used endogenously tagged Vps51-S-tag to pull-down GARP. Fig 10C displays colloidal Coomassie-stained gels corresponding to Vps54-S-tag (left) and Vps51-S-tag (right) samples. Only one band was differentially shifted by the presence of the S-tag, and MS/MS identified this band as Vps51. Vps51 was clearly more abundant in the pulled-down material of Vps51-S-tag cells, suggesting that Vps51 dissociates from the complex during purification. *S*. *cerevisiae* Vps51 is not critical for the assembly of the GARP complex but instead it is required for its stability [57]

Because *GARP* null mutations are similar in every phenotypic aspect, we chose *vps52Δ* to study if ChsB traffic is GARP-dependent. Like *rabCΔ*, *vps52Δ* diminished the amount of ChsB localizing to exocytic post-Golgi membranes (i.e. the SPK and the apical dome), and did so to a similar extent than *rabCΔ* (Fig 9D and 9E). This suggests that ChsB carriers transiting from endosomes to the TGN necessitate RabC/GARP to reach its destination efficiently. It also suggests that additional, GARP/RabC-independent pathways must contribute to deliver endocytosed ChsB to the TGN (see Discussion).

**Fig 11:**
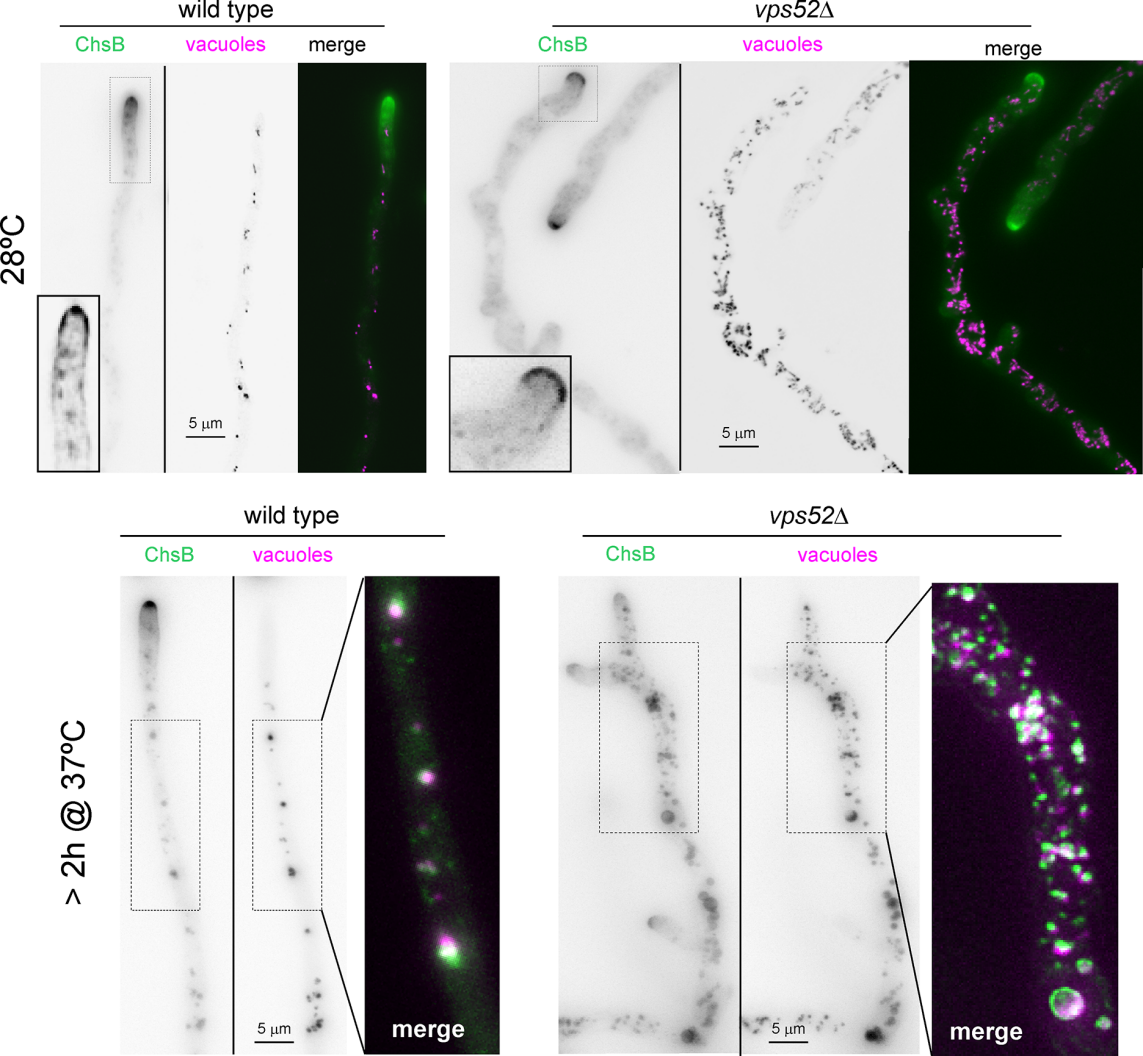
Delocalization of ChsB to the vacuolar system by *vps52Δ* Wt and *vps52Δ* strains expressing GFP-ChsB photographed at 28°C or following a shift to 37°C. Cells were stained with the vital dye CMAC (7-amino-4-chloromethylcoumarin) to reveal late endosomes and vacuoles (CMAC shown in magenta in color composites). The main images are MIPs of unprocessed z-stacks, but the insets were deconvolved to remove apical haze. Note that the contrast of the 28°C *vps52Δ* image has been adjusted to reveal the cytosolic haze, with ‘empty holes’ corresponding to the nuclei. Colocalization of ChsB with CMAC in the 37°C *vps52Δ* sample was complete.

The above microscopy experiments were conducted at 28°C. If endocytic cycling of ChsB and other CWMEs is central to hyphal growth, it should become more important at 37°C, in which the *A*. *nidulans* apical extension rate is highest. Thus, we shifted wt and *vps52Δ* cells to 37°C and examined the fate of ChsB, using CMAC staining to detect late endosomes and vacuoles. As noted above, the overall pattern of ChsB localization did not change in the wt shifted from 28°C to 37°C, even though ChsB localized to vacuolar structures in regions located away from the tip, reflecting its normal turnover (Fig 11). This picture was changed dramatically in the *vps52Δ* mutant, in which the internal ChsB pool was progressively displaced to the abundant vacuoles, whereas localization to exocytic post-Golgi membranes (i.e. the SPK and the apical dome PM) was virtually abolished. Indeed at 2 h after shifting cells to 37°C ChsB localized almost exclusively to vacuoles (Fig 11). Therefore, at 37°C, the capacity of GARP-independent pathways is insufficient to deliver ChsB to the apex and to rescue efficiently endosome-derived recycling carriers from endocytic degradation.

Taken together these results indicate that RabC/GARP-mediated endocytic recycling by way of the TGN is important to sustain the polarized delivery of ChsB to the growing apex, but that alternate pathway(s) capable of fulfilling this task, particularly when workload is relatively low, do exist.

## Discussion

**Fig 12:**
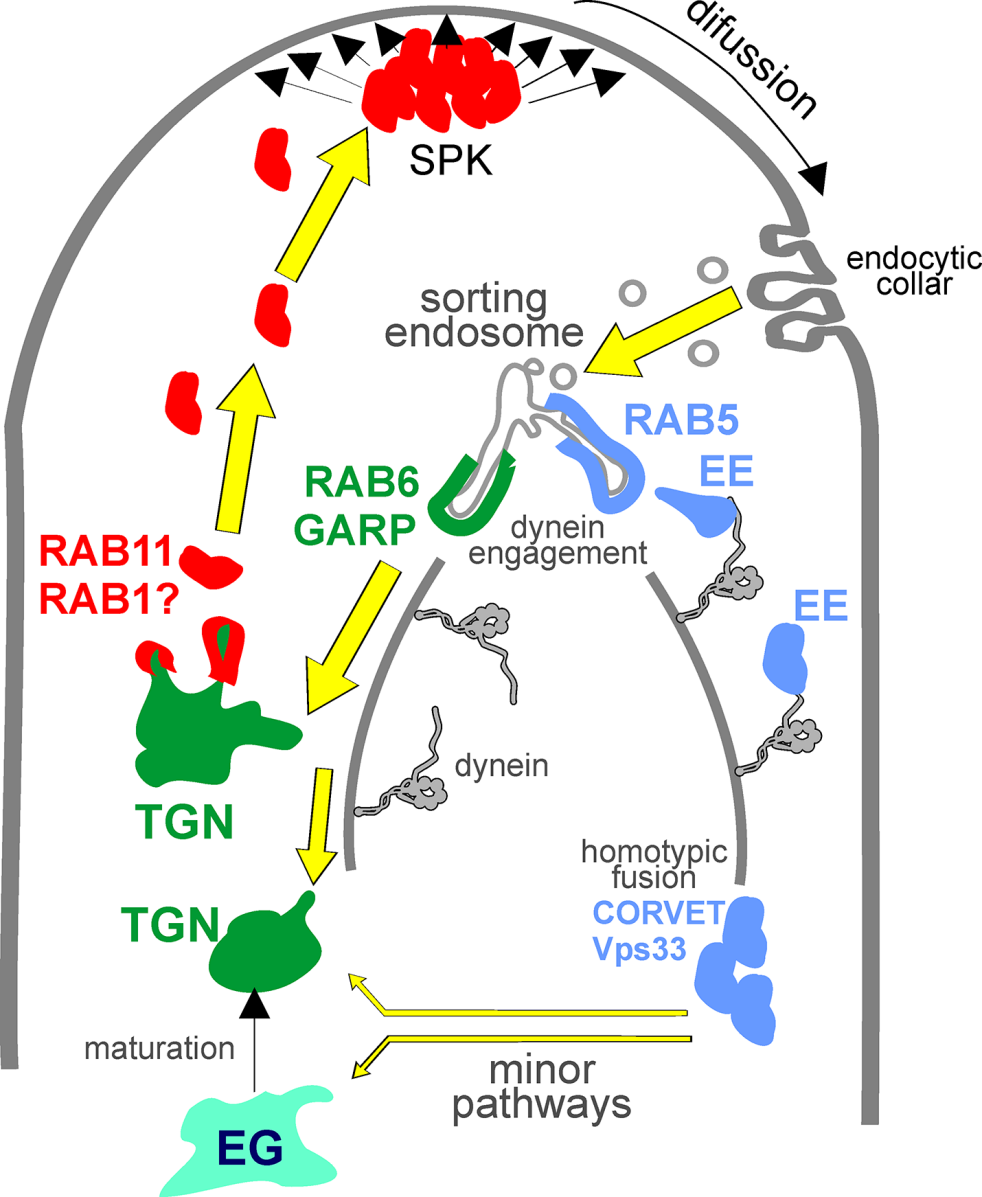
A model for ChsB recycling ChsB is transported with SVs (red) that accumulate at the SPK before being transported and tethered to the apical PM to undergo fusion. Once inserted into the PM ChsB undergoes diffusion away from the apex until it is captured and endocytosed by the subapical endocytic collar. Endocytic vesicles containing ChsB reach a mosaic of sorting endosomes. Here domains enriched in RabB^RAB5^ acquire EE identity (blue), engage dynein by means of the Hook complex and undergo basipetal transport and maturation across the degradative endocytic pathway. ChsB segregates into ‘recycling’ domains (green) that are delivered to the TGN in a RabC^RAB6^-, GARP- and dynein-dependent manner. Once at the TGN ChsB is selected into RabE^RAB11^ SVs (red), perhaps with cooperation of RabO^RAB1^, and delivered to the SPK. Alternative minor pathways (thinner yellow arrows) between degradative endosomes and either the TGN or the early Golgi (EG) that ensure the robustness of this crucial circuitry must exist, accounting for the proportion of ChsB that persists in the apical dome in the absence of RabC^Rab6^ /GARP.

Chitin synthases like ChsB participate in the synthesis of the fungal cell wall at sites of polarized growth. They act at the PM, using cytosolic uridine-diphospho-N-acetylglucosamine as substrate and extruding the resulting polymeric fibrils to the periplasm [15]. As integral membrane proteins, chitin synthases traffic towards the PM embedded into the membrane of SVs that, according to the currently accepted model are first transported to the SPK and subsequently undergo fusion with the PM [6–8](Fig 12). This two-stage model of traffic of SVs between the TGN and the PM has recently received strong support by experimental evidence revealing the existence of a negative correlation between the amount of SVs accumulating at the SPK and pulses of apical extension, implying that these pulses of growth correspond with the ‘discharge’ of SVs from the SPK [9]. Notwithstanding the fact that this mechanism ensures polarized delivery of ChsB, targeted exocytosis alone would be unable to generate polarity, as once delivered to the PM, ChsB would rapidly diffuse basipetally across the lipid bilayer, inappropriately extending its locale of action to regions far away from the delivery site. Work by Valdez-Taubas and Pelham (2003) establishing that locally exocytosed proteins polarize if they are endocytosed and recycled before they can diffuse to equilibrium inspired studies reporting that the endocytic machinery of *A*. *nidulans* predominates in a subapical collar that is appropriately located to act as a corral for proteins that function at the tip [17–19]. Well-established examples of *A*. *nidulans* proteins polarized by endocytic recycling are the R-SNARE SynA and the flippase DnfA^Dnf1^, for which endocytic sorting motifs whose mutational inactivation results in loss of polarization have been identified [14,21]. Notably, SynA polarization/endocytosis appears independent of the endocytic adaptor AP-2, contrasting with the complete dependence displayed by DnfA^Dnf1^ [58]. We hypothesized that a similar mechanism would restrict the activity of certain CWMEs to the hyphal tips. Here we demonstrate that ChsB is confined within a PM region (‘the apical dome’) located between the apex (the site of exocytosis) and the endocytic collar, and that impairment of endocytosis results in loss of polarization, consistent with ChsB undergoing endocytic recycling. Rather than recycling directly from endosomes [37] ChsB traverses through TGN cisternae, where it resides for ~1 min at 28°C before it is re-delivered to the SPK and the PM. Definitive evidence for ChsB following this indirect recycling pathway came from the observations that the enzyme is stranded in internal structures with TGN identity if Sec7 is acutely inactivated, and that the *geaA1* mutation that bypasses the role of Sec7 at the TGN [27] restores normal ChsB localization.

How does the enzyme, recycling by way of the TGN, return to the SPK/PM? In *A*. *nidulans* TGN membranes giving rise to SVs transit from Golgi to Post-Golgi identity following the recruitment of RabE^RAB11^ mediated by its GEF, TRAPPII [6,30]. The observation that the acute inactivation of Trs120, a key component of TRAPPII, relocalizes ChsB from the apical crescent to internal structures strongly suggests that ChsB follows this RAB11-dependent pathway, which would be consistent with the widespread involvement of RAB11 orthologues in endocytic recycling [59–63]. However, in *N*. *crassa* chitin synthases associate with a population of vesicles that localize to the central part (the ‘core’) of the SPK [64]. This core contains RAB1 (denoted here YPT-1) but not RAB11 = YPT-31), which instead associates with the peripheral layer. *A*. *nidulans* might differ from *N*. *crassa* in this regard, as RabE^RAB11^ does not show this stratification [6], even though RAB1 is also present at the SPK (besides the Golgi) [26], where it colocalizes with ChsB. Of note, work in *S*. *cerevisiae* has shown that RAB1 (Ypt1p) and RAB11 (Ypt31) cooperate to regulate Sec7 in the TGN [41].

### The dynein requirement

The fact that inactivation of dynein results in ChsB accumulation in a subapical membranous compartment readily accessible to FM4-64 (Fig 5) strongly supports the existence of an endosomal compartment through which ChsB transits in route between the PM and the TGN. EEs, which contain and require RAB5 for function, move away from the tip using dynein, but are unlikely to mediate this route because ChsB is normally polarized in a mutant in which the major RAB5 paralogue is ablated (Fig 9B), supporting the contention that ChsB originates from an endosome that lies upstream of, and is functionally different from RAB5 endosomes, and that dynein would be required to connect this with the TGN (Fig 12). However, because dynein-mediated movement is linked to the maturation of EEs [43,65], it is possible that the endosome membranes at which ChsB is stranded overlap with the aggregate of membranes resulting from the inability of EEs to move away from the tip [20,47,66–69]. In this scenario, the absence of pulling force due to lack of dynein might prevent two different domains of a sorting endosome, destined for recycling to the TGN and for endocytic RAB5-dependent degradation, respectively, from being resolved (Fig 12), stalling ChsB in an abnormal compartment of mixed identity. Notably SynA is also present in this abnormal endosome [20], further underlining the similarities between ChsB and SynA trafficking.

### The SynA pathway and RabC/GARP

SynA forms SNARE bundles containing Vti1, TlgA^Tlg1^ and TlgB^Tlg2^ (the TGN syntaxin) [42], potentially mediating fusion between membranes deriving from the above sorting endosome and the TGN. Paradoxically a double *tlgAΔ tlgBΔ* mutant is only slightly impaired in SynA localization, indicating that recycling to the TGN can occur by an alternative pathway. Co-Immunoprecipitation experiments suggested that this pathway involves SedV^Sed5^ [42] (see also below).

Efficient transport from sorting endosomes to the TGN requires RabC^RAB6^ acting in its capacity to recruit the GARP complex that tethers vesicles to the TGN [52]. Null GARP mutations result in a major growth defect, underlining the key role that recycling to the TGN plays in the physiology of hyphae. These mutations or *rabCΔ* incompletely delocalized ChsB from the tip at 28°C. However, complete ChsB delocalization took place after shifting cells to 37°C, and this delocalization must result from failure to recycle to the TGN from endosomes as it occurs in parallel with ChsB diversion towards vacuoles. The apical extension rate is highest at 37°C, in all likelihood imposing a heavier workload on recycling.

A possible ancillary factor of the tethering function of GARP, potentially accounting for the ChsB remaining at the apical dome of GARP mutants at 28°C, is the conserved oligomeric Golgi (COG) complex [70], which can act both at the TGN [71], cooperating with syntaxin-6 (TlgB^Tlg2^), and at the early Golgi, cooperating with syntaxin-5 (SedV^Sed5^) [72–74]. It is also possible that in the absence of GARP a proportion of ChsB recycles directly from the sorting endosome [37]. However, the possibility that the biosynthesis of ChsB is augmented in GARP mutants to compensate for the recycling defect seems unlikely, as the steady state levels of ChsB are unaltered in a *vps52Δ* mutant (S5 Fig). Thus our data suggest that alternate pathways can maintain ChsB polarization, ensuring the robustness of endocytic cycling, which seems crucial for rapid apical extension. Among these pathways, the GARP-mediated one appears to be dominant, such that at 37°C, the needs for recycling imposed by rapid apical growth can scarcely be met in strains lacking GARP. Thus the cycling route followed by ChsB (and SynA) would be akin to that followed by Snc1 in *S*. *cerevisiae*, which is Ypt6 (RabC^RAB6^)-dependent [75]. Of note, the recycling of the *A*. *nidulans* flippase DnfA^Dnf1^ also appears to be Vps54 dependent [14]

In view of the debilitating growth phenotype of GARP mutations, we speculate that besides ChsB other cargoes important for the hyphal mode of growth circulate through this endocytic cycling pathway. Polarity landmarks are alluring hypothetical candidates, as suggested by the instability of polarity axes resulting from down-shifting SlaB^Sla2^ expression. In *S*. *cerevisiae* Cdc42 has been shown to polarize by endocytic recycling, corralled by endocytosis at the site of bud growth. Cdc42 recruits formin, polarizing actin cables, and thus transport of SVs, leading to positive feedback [76,77]. Notably yeast *sla2Δ* results, like *A*. *nidulans* SlaB^Sla2^ downregulation, in a multiplicity of polarity axes [77]. Phosphatidylserine transported with SVs helps to stabilize Cdc42 [78], highlighting the potential role of lipids. However we note that polarity determination in *A*. *nidulans*, which like many other fungi contains both Cdc42 and Rac1 orthologues that show functional overlap [79], is likely to be more complex. Moreover, microtubules, in addition to actin cables, transport SVs to the apex, mediating transport of other polarity landmarks such as TeaR to the cell surface [80].

Additional potential cargoes for endocytic cycling are other CWMEs. In *U*. *maydis* two chitin synthases and the 1,3-β-glucan synthase travel in the same secretory vesicle, hypothetically facilitating cell wall synthesis [81]. In contrast, in *N*. *crassa* 1,3-β-glucan synthase and chitin synthases appear to reside in different types of secretory vesicles [10,12]. Future work will address whether other integral membrane CWMEs are co-passengers with ChsB in the recycling pathway studied here. We should note that the ChsB/GARP pathway, critical for apical extension, is a possible target for antifungal intervention. In this regard a homozygous *C*. *albicans arl1Δ* lacking a GTPase cooperating with RAB6 in the tethering of endosome-derived vesicles to the TGN is deficient in filament formation and drastically reduced in virulence [82]

## Materials and methods

### *Aspergillus* techniques and strains

Complete (MCA) and synthetic complete (SC) media [83] containing 1% glucose and 5 mM ammonium (+)-tartrate as carbon and nitrogen source, respectively, were routinely employed. Strains used in this work are listed in S1 Table. Deletion alleles of *chsB*, *vps51*, *vps52*, *vps53* and *vps54* were obtained by transformation, using PCR-assembled constructs [84] (primers detailed in S2 Table).

### Fluorescent protein tagging

GFP-ChsB and mCh-ChsB were expressed from N-terminally tagged versions of *chsB* constructed by gene replacement after transformation with PCR-assembled constructs (primers in S2 Table). For mCh-ChsB we used a ‘5-way PCR’ cassette [18] consisting of 916 bp of *chsB* ‘distal’ upstream region (primers MHG231 and MHG232), 1900 bp encoding *A*. *fumigatus pyrG* (MHG233 and MHG234), 978 bp of *chsB* ‘proximal’ upstream (promoter) region plus 5’-UTR (MHG235 and MHG236), 738 bp encoding mCh-(Gly-Ala)5 (MHG262 and MHG261) and 2171 bp encoding the N-terminal region of ChsB starting at the initiation Met codon (MHG239 and MHG174). The 6709 bp codon-adapted GFP-ChsB cassette was similar but contained a 744 fragment encoding GFP-(Gly-Ala)5 (MHG237 and MHG238) instead of the mCh-(Gly-Ala)5 fragment. Gene replacement events were genotyped by Southern blotting. Another version of the N-terminal GFP cassette, engineered for riboflavin auxotrophy selection instead of pyrimidine selection contained a 1971 bp fragment encoding *A*. *fumigatus riboB* (MHG282 and MHG283) instead of the corresponding *pyrG*^*Af*^ fragment.

The cassette for C-terminal GFP tagging consisted of a 4633 gene replacement DNA fragment including, in sequential order, 900 bp of the *chsB* C-terminal coding region (MHG144 and MHG145), 1900 bp encoding *A*. *fumigatus pyrG* as above, 744 bp encoding (Gly-Ala)5-GFP (MHG146 and MHG147), 289 bp of 3’-UTR (MHG148 and MHG149), and a further 800 bp of *chsB* downstream DNA (MHG152 and MHG153). The cassette for ChsB-mCh was similar excepting the fluorescent protein tag, which consisted of a 744 bp fragment encoding (Gly-Ala)5-mCherry (MHG246 and MHG271). Gene replacement events were genotyped by Southern blotting.

### General microscopy techniques and image acquisition

These have been described in detail [3]. Briefly hyphae were cultured in pH 6.8 ‘watch minimal medium’ (WMM) using 8-well chambers (IBIDI GmbH, Martinriesd, Germany). Images were acquired with a Leica DMI6000 B inverted microscope equipped with a Leica 63x/1.4 N.A. Plan Apochromatic objective and a Hamamatsu ORCA ER digital camera (1344 x 1024 pixels) using, for single channel acquisition, Semrock GFP-3035B and TXRED-4040B ‘BrightLine’ filter cubes. For simultaneous channel acquisition a Dual-View beam splitter (Photometrics) equipped with the supplier’s filter sets for GFP and mCherry fluorescence channels was used, except for the experiment shown in the S2 Movie, which was performed with a Hamamatsu ORCA Flash 4.0 CMOS camera coupled to a Gemini beam splitter (Hamamatsu) with appropriate filters. The incubation temperature for microscopy cultures was normally 28°C. When the incubation temperature was shifted from 28°C to 37°C [26] cultures reached the target within 15–20 min. To determine the effects of *vps33*^*ts*^ on ChsB, cultures were shifted to 42°C for 90 min in an external incubator, transferred to the microscope chamber pre-warmed at 37°C and photographed immediately. FM4-64 (Molecular Probes, 1 μM) was loaded for 5 min before washing with fresh medium [39]. CMAC (Molecular Probes, 10 μM) was loaded for 5 min followed by three washes with fresh medium [65]. Latrunculin B was used at a final concentration of 0.1 mM [28].

For shift-down experiments involving SlaB^Sla2^ downregulation, *slaB1* and wt conidia were inoculated in WMM containing 0.05–0.1 mM nitrate as sole N source. Microscopy chambers were first incubated at 26°C for 10–11 h. At this point the medium was removed and substituted by WMM containing 40 mM ammonium chloride (after three washes with the same medium). These secondary cultures were incubated for a further 12 h before being photographed (GFP-ChsB). Control cultures correspond to cells kept continuously cultured in nitrate WMM.

### Image manipulation and analyses

All images were processed using Metamorph 7.7.0. Once converted to 8-bit greyscale or 24-bit RGB they were annotated with Corel Draw (Corel, Ottawa, Canada). Single channels are usually shown in inverted greyscale. When indicated, Z-stacks were deconvolved with Huygens Professional (Scientific Volume Imaging, Hilversum, the Netherlands, EU). GraphPad Prism 7.03 (GraphPad software) and SigmaPlot 12.5 (Systat Software) were used for statistical analysis (detailed in Fig legends) and graphical display of datasets. Movies were built with Metamorph. Annotated movies were converted to QuickTime using Image J, and file size was adjusted using Sorenson or mpeg-4 compression.

For colocalization analyses we used maximal intensity projections (MIPs) of deconvolved Z-stacks. Following channel alignment with Metamorph, images were further processed with Image J (WCIF 1.37c). Li’s and Pearson’s colocalization coefficients [85] were determined for ROIs (drawn with ‘freehand selection’) that covered the complete width of the hyphae excluding the ‘empty’ regions corresponding to the nuclei, which are devoid of Golgi cisternae. Li’s coefficients [85] were calculated with the ‘intensity correlation analysis’ plugin. The resulting individual data were exported to Microsoft Excel and used to obtain normalized intensity values, setting the maximal value to 1. Pearson’s coefficients for the above regions were calculated with the Coloc2 colocalization plugin of FIJI.

To demonstrate that apex-proximal TGN cisternae are enriched in ChsB, intensities of GFP-ChsB y mRFP-PH^OSBP^ signals in 60 TGN cisternae located within the apicalmost 10 μm were determined from maximal intensity projections of z-stacks. Intensity values were plotted *vs*. distance to the tip. Datasets were analyzed with SigmaPlot to obtain the Pearson Product Moment Correlation coefficients (*r*) and the corresponding *P* values.

To estimate the amount of polarized GFP-ChsB in wt, *rabCΔ* and *vps52Δ* strains, middle planes of z-stacks were used to determine fluorescence intensities along 50 px-long and 2 px wide ROIs covering the plasma membrane in the apical domes (with Metamorph). Data were plotted as average pixel intensities.

### GARP purification

Briefly, mycelia were harvested from fungal cultures made with minimal medium supplemented with 2.5% (v/v) corn steep liquor (Solulys 048R, Roquette Laisa S.A., Spain), and containing 20 mM ammonium sulfate and 3% sucrose (w/v) as main nitrogen and carbon sources, respectively. Lyophilized mycelia (2 g dry weight) were ground with a ceramic bead in a FastPrep bead beater. The resulting powder was mixed with 50 ml of 25 mM HEPES, pH 7.5, 0.5% IGEPAL, 300 mM KCl, 2 mM EDTA, 1 mM DTT, 2 M MG132 and Complete ULTRA EDTA-free protease inhibitor cocktail (Roche). The suspension was next homogenized with glass beads (0.6 mm) in the FastPrep for 15 sec at maximal power, and further incubated for 10 min at 4°C in a rotating wheel. This procedure was repeated twice before clarifying the extract by centrifugation at 15,000 x g for 30 min. The supernatant was mixed with 500 μl of S-protein Agarose beads (Novagen) (40 μl of packed beads per 100 mg of protein) and incubated for 4 h at 4°C in a rotating wheel in the presence of BSA 1% (w/v). Beads were next washed four times with 10 ml of washing buffer (25 mM HEPES pH 7.5, 300 mM KCl, 2 mM EDTA and 1 mM DTT) for 10 min at 4°C. Proteins were eluted after boiling beads in Laemmli buffer or, for MS/MS determinations, after incubation for 15 min at 37°C with 500 μl of 10 mg/ml of S-peptide (KETAAAKFERQHMDS), adjusted at pH 7.5. Proteins were trichloroacetic acid-precipitated, resuspended in Laemmli buffer and resolved by SDS-PAGE followed by silver staining for analytical purposes or, for MS/MS of bands excised from the gels, with colloidal Coomassie. MS/MS and band identification were as described [30].

## Supporting information

S1 Fig mCh-ChsB and GFP-ChsB comparison. Top, merge of MIPs of deconvolved z-stacks of a mixed culture of gene-replaced mCh-ChsB and GFP-ChsB strains. Bottom, growth phenotype of the indicated gene-replaced strains is compared to the wt.
(PDF)

S2 Fig Colocalization analyses of mCh-ChsB and GFP-TlgB^Tlg2^ in a *hypB5* (*sec7*^*ts*^) strain. Pictures taken at different times after shifting cells at 37ºC, with indication of Pearson’s and Li’s colocalization coefficients. Plots were used to calculate Li’s ICQ.
(PDF)

S3 Fig CMAC staining of vacuoles in hyphae of strains with the indicated phenotypes. (PDF)

S4 Fig Abnormal morphology and depolarization of TGN cisternae resulting from GARP mutations. Cisternae of the TGN were labeled with PH^OSBP^. Boxed regions were magnified 2.5 times in the right insets. Arrows point at examples of typically fenestrated cisternae that are not seen in the mutants. For linescans, 1 px = 0.103 μm.
(PDF)

S5 Fig Levels of GFP-ChsB are similar in wt and *vps52Δ* cells. Anti-GFP was used to detect GFP-ChsB. Anti-PSTAIR antibody (AbCam) was used for the loading control. This antibody detects a conserved epitope present in cyclin-dependent kinases. In *A*. *nidulans* it reacts with PhoA (41.3 kda), NimX^Cdc2^ (36.8 kDa) and PhoB (35.9 kDa) cyclin-dependent kinases.
(PDF)

S1 Table Strains used in this work. (PDF)

S2 Table Oligonucleotides used for genetic manipulations and diagnostic PCRs. (PDF)

S1 Movie GFP-ChsB and mCh-AbpA move forward concertedly as hyphal tip growth proceeds. Frames are maximal intensity projections of z-stacks acquired every 2 min over an 80 min time period. Time scale in min:sec.
(MOV)

S2 Movie mCh-ChsB and GFP-RabE^RAB11^ colocalize at the SPK over time. Frames are middle planes acquired every sec for a 4 min period, using a Gemini beam splitter. The movie is accelerated 15 times. Time scale in min:sec.
(MOV)
