# Supplementary material for: Endocytic recycling via the TGN underlies the polarized hyphal mode of life
Source: PLoS Genet. 2018 Apr 2;14(4):e1007291. doi: 10.1371/journal.pgen.1007291 (PMC5880334; doi:10.1371/journal.pgen.1007291)
Supplement: S1 Fig — Top, merge of MIPs of deconvolved z-stacks of a mixed culture of gene-replaced mCh-ChsB and GFP-ChsB strains. Bottom, growth phenotype of the indicated gene-replaced strains is compared to the wt. (PDF) [file pgen.1007291.s001.pdf]

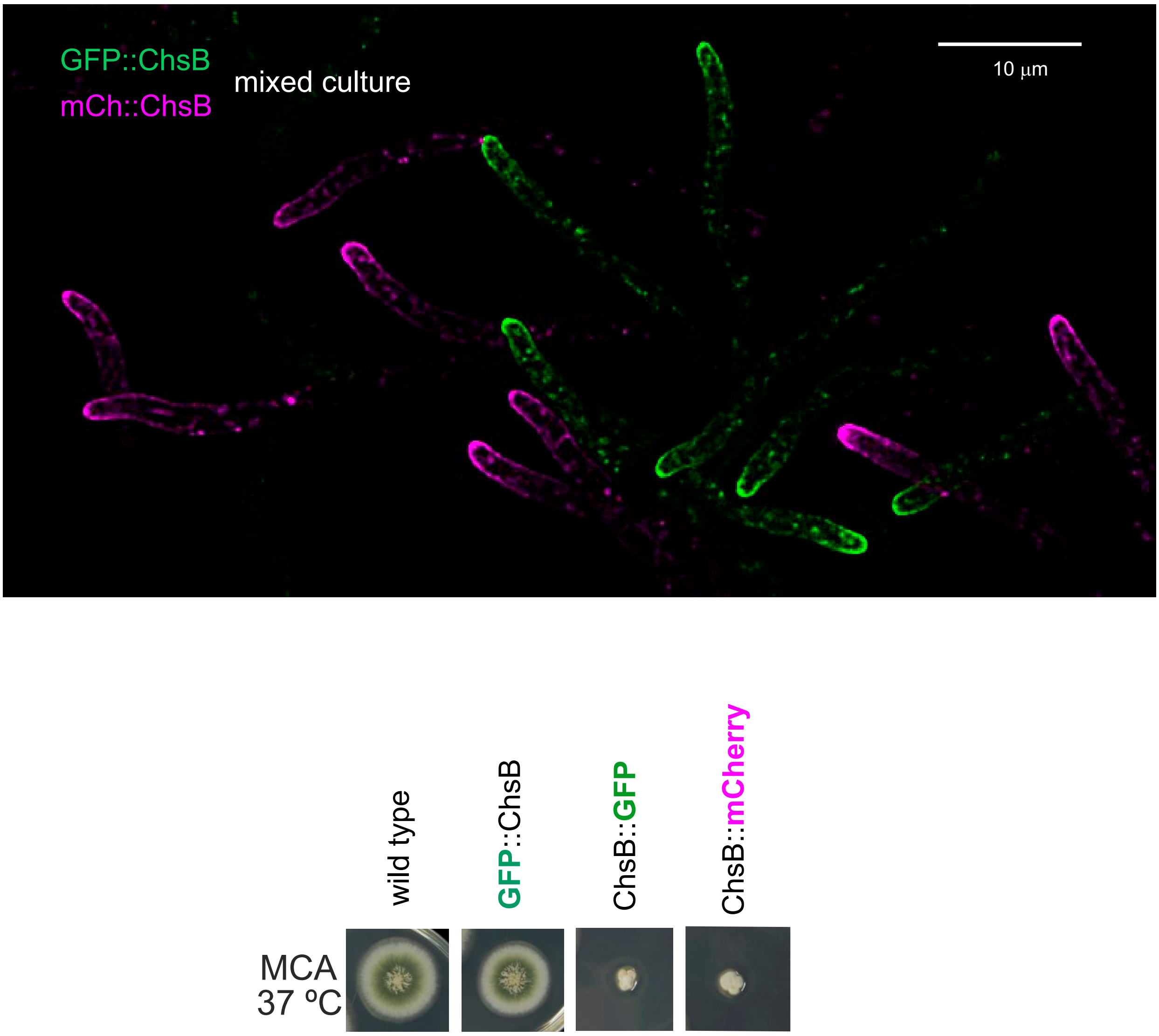

S1 Figure: mCh-ChsB and GFP-ChsB comparison
Top, merge of MIPs of deconvolved z-stacks of a mixed culture of gene-replaced mCh-ChsB and GFP-ChsB strains. Bottom, growth phenotype of the indicated gene-replaced strains is compared to the wt.
