# Supplementary material for: Endocytic recycling via the TGN underlies the polarized hyphal mode of life
Source: PLoS Genet. 2018 Apr 2;14(4):e1007291. doi: 10.1371/journal.pgen.1007291 (PMC5880334; doi:10.1371/journal.pgen.1007291)
Supplement: S2 Fig — Pictures taken at different times after shifting cells at 37ºC, with indication of Pearson’s and Li’s colocalization coefficients. Plots were used to calculate Li’s ICQ. (PDF) [file pgen.1007291.s002.pdf]

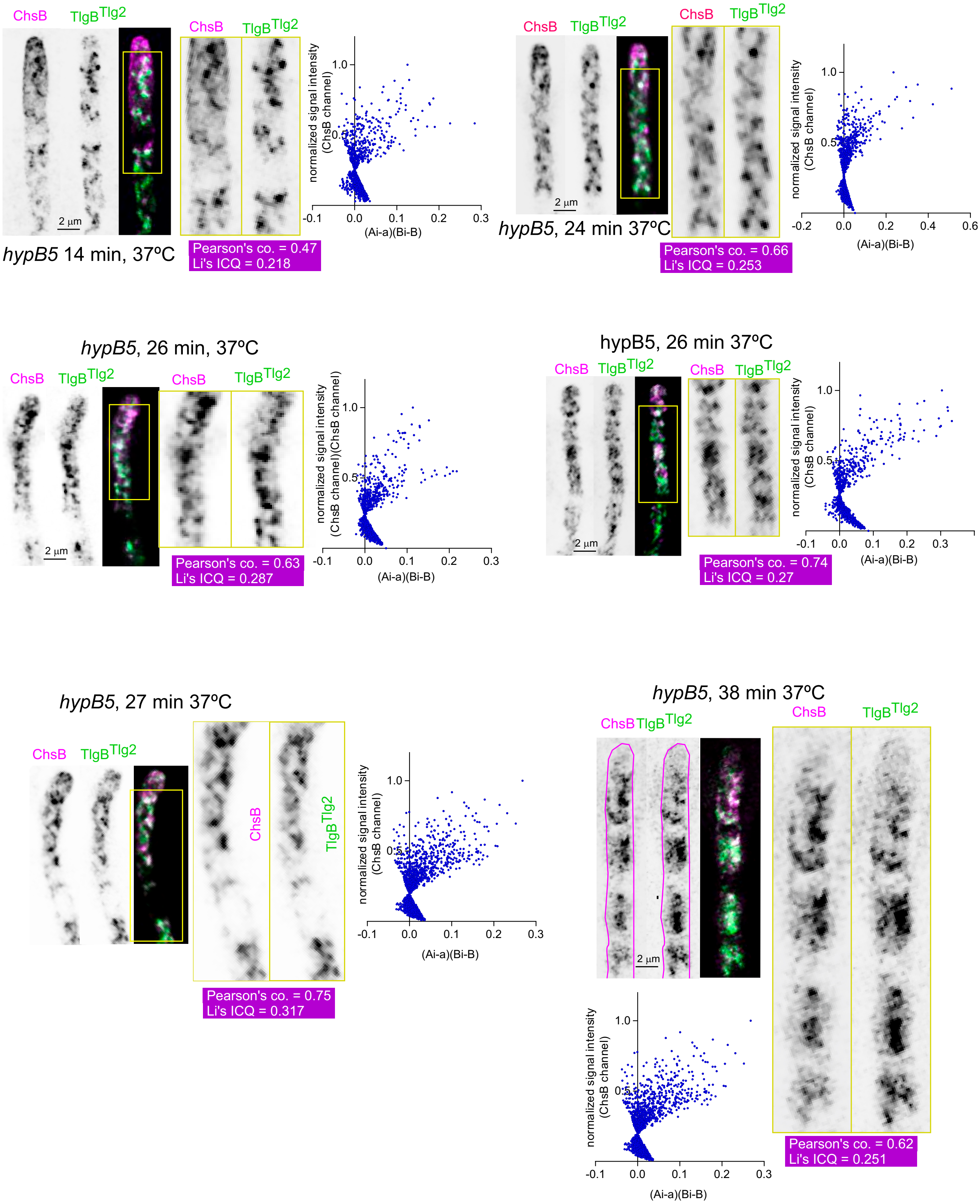

S2 Figure. Colocalization analyses of mCh-ChsB and GFP-TlgB^{Tlg2} in a hypB5 (sec7^{ts}) strain
Pictures taken at different times after shifting cells at 37ºC, with indication of Pearson's and Li's colocalization coefficients. Plots were used to calculate Li's ICQ.
