# Supplementary figures and images for: Endocytic recycling via the TGN underlies the polarized hyphal mode of life
Source: PLoS Genet. 2018 Apr 2;14(4):e1007291. doi: 10.1371/journal.pgen.1007291 (PMC5880334; doi:10.1371/journal.pgen.1007291)

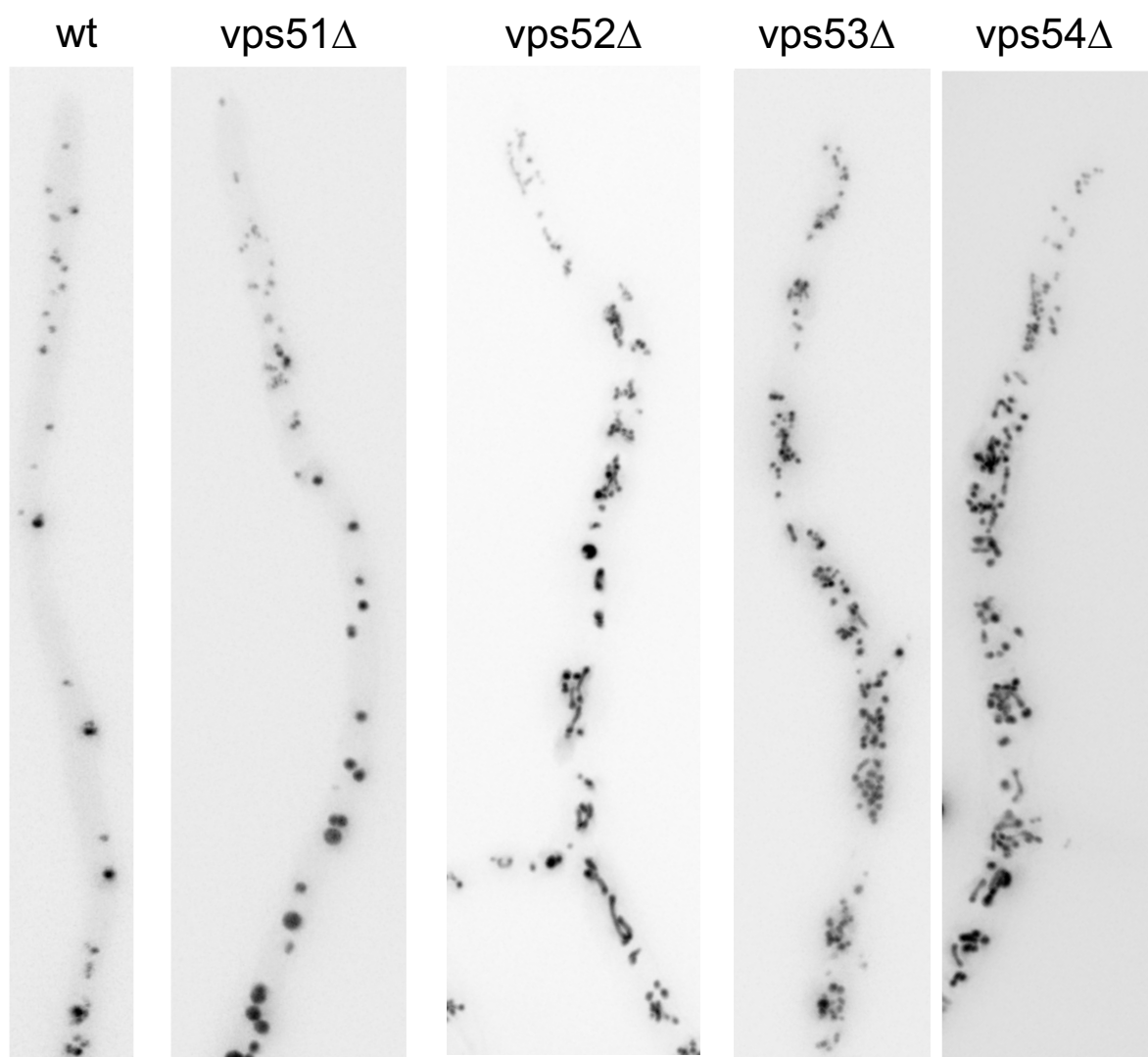

**S3 Figure. CMAC staining of vacuoles in hyphae of strains with the indicated genotypes.**

Supplement: S3 Fig — (PDF) [file pgen.1007291.s003.pdf]
