# Supplementary material for: Endocytic recycling via the TGN underlies the polarized hyphal mode of life
Source: PLoS Genet. 2018 Apr 2;14(4):e1007291. doi: 10.1371/journal.pgen.1007291 (PMC5880334; doi:10.1371/journal.pgen.1007291)
Supplement: S4 Fig — Cisternae of the TGN were labeled with PHOSBP. Boxed regions were magnified 2.5 times in the right insets. Arrows point at examples of typically fenestrated cisternae that are not seen in the mutants. For linescans, 1 px = 0.103 μm. (PDF) [file pgen.1007291.s004.pdf]

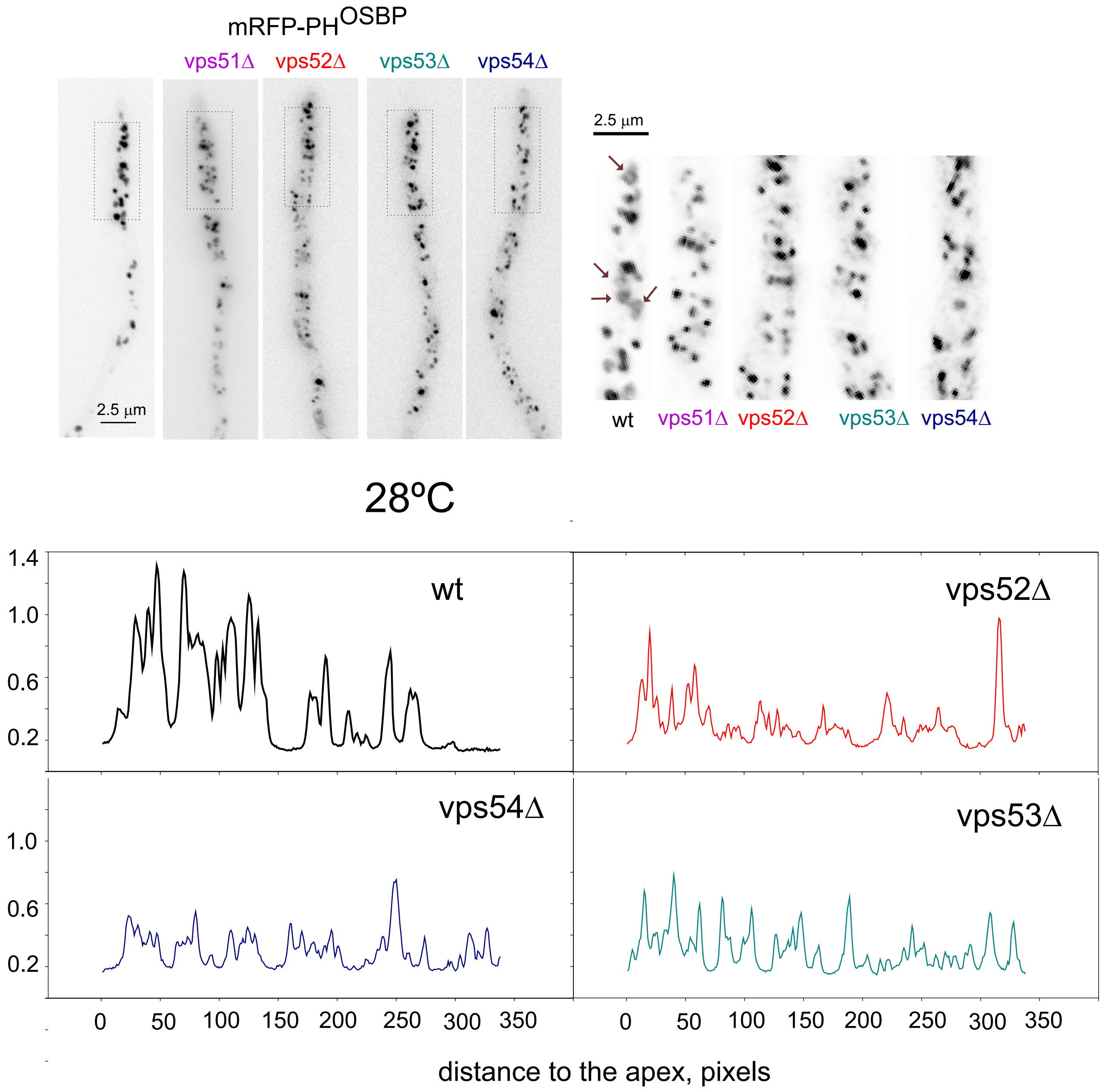

S4 Figure. Abnormal morphology and depolarization of TGN cisternae resulting from GARP mutations. Cisternae of the TGN were labeled with PH^{OSBP}. Boxed regions were magnified 2.5 times in the right insets. Arrows point at examples of typically fenestrated cisternae that are not seen in the mutants. For linescans, 1 px = 0.103 µm
