# Supplementary material for: Endocytic recycling via the TGN underlies the polarized hyphal mode of life
Source: PLoS Genet. 2018 Apr 2;14(4):e1007291. doi: 10.1371/journal.pgen.1007291 (PMC5880334; doi:10.1371/journal.pgen.1007291)
Supplement: S5 Fig — Anti-GFP was used to detect GFP-ChsB. Anti-PSTAIR antibody (AbCam) was used for the loading control. This antibody detects a conserved epitope present in cyclin-dependent kinases. In A. nidulans it reacts with PhoA (41.3 kda), NimXCdc2 (36.8 kDa) and PhoB (35.9 kDa) cyclin-dependent kinases. (PDF) [file pgen.1007291.s005.pdf]

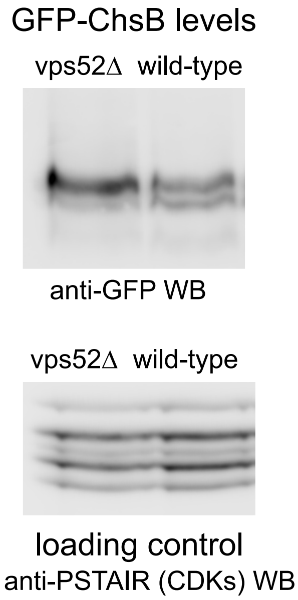

S5 Figure. Levels of GFP-ChsB are similar in wt and *vps52*Δ cells.
Anti-GFP was used to detect GFP-ChsB. Anti-PSTAIR antibody (AbCam) was used for the loading control. This antibody detects a conserved epitope present in cyclin-dependent kinases. In A. nidulans it reacts with PhoA (41.3 kda), NimX^{Cdc2} (36.8 kDa) and PhoB (35.9 kDa) cyclin-dependent kinases.
