# Supplementary material for: Endocytic recycling via the TGN underlies the polarized hyphal mode of life
Source: PLoS Genet. 2018 Apr 2;14(4):e1007291. doi: 10.1371/journal.pgen.1007291 (PMC5880334; doi:10.1371/journal.pgen.1007291)
Supplement: S1 Table — (PDF) [file pgen.1007291.s006.pdf]

Table S1. *A. nidulans* strains used in this work.

MAD number	Genotype	Source
2	biA1	Our collection
3579	wA4; pyroA4[pyroA*-gpdA^{mini}::mrfp-PH^{OSBP}]	Pantazopoulou *et al.* 2009
5854	pyrG89; nkuAΔ::bar pyroA4; pyrG^{Af}::chsBprom-chsB	This work
5559	pyrG89; pyroA4 nkuAΔ::bar; chsB-gfp::pyrG^{Af}	This work
5626	pyrG89; nkuAΔ::bar pyroA4; chsB-mCherry-3'UTR-pyrG^{Af}	This work
5851	pyrG89; nkuAΔ::bar pyroA4; pyrG^{Af}::chsBprom::gfp-chsB	This work
5895	pyrG89; nkuAΔ::bar; pyroA4[gpdAmini::mRFP::PH^{OSBP}]; pyrG^{Af}::gfp-chsB	This work
5897	pyrG89? hypA1 pabaA1; nkuAΔ::bar?; pyrG^{Af}::chsBprom::gfp-chsB	This work
5924	pyrG89 pabaA1 yA2; nkuAΔ::bar?; pyrG^{Af}::gfp-chsB abpA-mRFP::pyrG^{Af}	This work
5928	pyrG89; nkuAΔ::bar pyroA4; pyrG^{Af}::chsBprom::mCherry-chsB	This work
5981	pyrG^{Af}::mCherry-chsB rabEp::rabE::rabEp::gfp-rabE::pyrG^{Af} pyrG89? nkuAΔ:: bar? pyroA4 pantoB100	This work
6058	pyrG^{Af}::ChsBprom::mCherry-chsB pyrG^{Af}::GFP-synA pyroA4 pyrG89? nkuAΔ::bar?	This work
6116	pyrG89?; nkuAΔ:: bar?; pantoB100 vps33^{ts} pyrG^{Af}::gfp-chsB	This work
6222	pyrG89; nkuAΔ:: bar rabCΔ::riboB^{Af}; pyrG^{Af}-gfp-chsB; riboB2	This work
6253	pyrG89? hypB5; nkuAΔ::bar?; pyroA4; pyrGAf::chsBprom-mCherry-chsB; geaA1-gfp	This work
6268	pyrG89?;argB2[argB*-alcA-gfp-rabE]?; nkuAΔ::bar?; pyroA4::[pyroA^{t}-gpda^{mini}-gfp-tlgB]; pyrG^{Af}::chsBprom::mCherry-chsB	This work
6269	pyrG89? hypB5;argB2[argB*-alcA-gfp-rabE]?; nkuAΔ::bar?; pyroA4::[pyroA^{t}-gpda^{mini}-gfp-tlgB]; pyrG^{Af}::chsBprom::mCherry-chsB	This work
6327	pyrG89; riboB^{Af}-prom-gfp-chsB; nkuAΔ::bar pyroA4; riboB2	This work
6402	fimAΔ::tn31::pyr4 pyrG89?; nkuAΔ::bar?; pyrG^{Af}::chsBprom::gfp-chsB pantoB100	This work
6422	pyrG89; vps52::pyrG^{Af}; nkuAΔ::bar pyroA4; riboB^{Af}-prom-gfp-chsB; riboB2	This work
6510	pyrG89?; nkuAΔ::bar inoB2 pyroA4[pyroA*-gpdAsh::mCherry::sed5]; pyrG^{Af}::chsBprom-gfp-chsB niiA4	This work

MAD number	Genotype	Source
6511	*pyrG89*; *riboBAf-prom-gfp-chsB; nkuA*Δ*::bar* pyroA4; *:pyrG*^{Af}*::niiAp::slaB*; *riboB2*	This work
6552	*pyrG89?*; *nkuAΔ::bar? pyroA4; pyrG*^{Af}*::chsBprom-gfp-chsB;* *nudA2 chaA1*	This work
6553	*pyrG89?*; *wA3*; *nkuA*Δ::bar? *pyroA4*; *pyrG*^{Af}::*chsBprom-gfp-chsB* ; *nudA5*	This work
6559	*pyrG89? pabaA1*; *rabB*Δ::*riboB*^{Af}*; nkuA*Δ::*bar? pyroA4*; *inuA-HA-3'UTR-pyrG*^{Af}*? riboB2?*; *pyrG*^{Af}::*chsBprom-gfp-chsB*	This work
6290	*pyrG89; pyroA4 nkuA*Δ*::bar; vps51*Δ::*pyrG*^{Af}*; riboB2*	This work
5698	*pyrG89; vps52*Δ::*pyrG*^{Af}*; pyroA4 nkuA*Δ*::bar; riboB2*	This work
6501	*pyrG89; pyroA4 nkuAΔ::bar; vps53Δ::pyrG*^{Af}*; riboB2*	This work
5795	*pyrG89; vps54Δ::pyrG*^{Af}*; pyroA4 nkuAΔ::bar; riboB2*	This work
6341	*pyrG89; vps54-Stag::pyrG*^{Af}*; pyroA4 nkuAΔ::bar; riboB2*	This work
6503	*pyrG89; pyroA4 nkuAΔ::bar; vps51-Stag::pyrG*^{Af}*; riboB2*	This work
6467	*pyrG89; wA4 vps52Δ::pyrG*^{Af}*; pyroA4*[*pyroA*-gpdA*^{mini}*::mrfp-PH*^{OSBP}] *nkuAΔ::bar; riboB2*	This work
6469	*pyrG89; wA4; pyroA4*[*pyroA*-gpdA*^{mini}*::mrfp-PH*^{OSBP}] *nkuAΔ::bar; vps53Δ::pyrG*^{Af}*; riboB2*	This work
6471	*pyrG89; wA4 vps54Δ::pyrG*^{Af}*; pyroA4*[*pyroA*-gpdA*^{mini}*::mrfp-PH*^{OSBP}] *nkuAΔ::bar; riboB2*	This work
6494	*pyrG89; wA4;* *pyroA4*[*pyroA*-gpdA*^{mini}*::mrfp-PH*^{OSBP}] *nkuAΔ::bar; vps51Δ::pyrG*^{Af}*; riboB2*	This work

**Strain numbers refer to the MAD (Madrid, CIB-CSIC) collection.*
