# Supplementary material for: Endocytic recycling via the TGN underlies the polarized hyphal mode of life
Source: PLoS Genet. 2018 Apr 2;14(4):e1007291. doi: 10.1371/journal.pgen.1007291 (PMC5880334; doi:10.1371/journal.pgen.1007291)
Supplement: S2 Table — (PDF) [file pgen.1007291.s007.pdf]

Table S2. Oligonucleotides used for genetic manipulations and diagnostic PCRs

Code	Name	5’-3’ Sequence
MHG_144	Fw chsB orf medio	TACATCTACCTTGCGTTCCTGC
MHG_145	Rev chsB final	CCGACGGGCGAAGCAGCAG
MHG_146	Fw fus chsB-gfp	CTGCTGCTTCGCCCGTCGGGGAGCTGGTGCAGGCGCTGGAGCC
MHG_147	rev fus gfp-3utr chsB	CCATTAGGCAAAGCATGCTCTTATTATTTGTATAGTTCATCCATGCC
MHG_148	Fw 3'utr chsB	TAAGAGCATGCTTTGCCTAATGG
MHG_149	rev chsB final transcrip	TACAGTACGGGATTAGAGTAAAG
MHG_150	fw fus chsB dwn-pyrG	CTTTACTCTAATCCCGTACTGTAACCGGTCGCCTCAAACAATGCTC
MHG_151	rev fus pyrG-down chsB	CTTGTACACAAGCCATCGCCTCCTGTCTGAGAGGAGGCACTGATGCG
MHG_152	fw chsB down	GAGGCGATGGCTTGTGTACAAG
MHG_153	rev chsB down	GGTAAATCTTTTGCGTGCACTTG
MHG_166	fw chsB up	TTGGTGCTCAATAAGATTGGCTG
MHG_167	rev chsB up	GGTTAAACTGGTAGTATGTGCG
MHG_168	fw fus up chsB-pyrG	CGCACATACTACCAGTTTAACCACCGGTCGCCTCAAACAATGCTC
MHG_169	rev fus down-chsB pyrG	CTTTCCATTAGGCAAAGCATGCTCCTGTCTGAGAGGAGGCACTGATGCG
MHG_170	fw chsB up	GAGCATGCTTTGCCTAATGGAAAG
MHG_171	rev fus mcherry-3utr chsB	CCATTAGGCAAAGCATGCTCTTATTACTTGTACAGCTCGTCCATGC
MHG_174	rev medio orf chsB	CAAGGATAAACTGCAAGAGCAGG
MHG_231	Fw upstream real chsB	AAGAATAGGAACAAAGACCGCTAG
MHG_232	Rev up chsB	CGGCCTGGCGGTAATTTAGTTC
MHG_233	Fw fus up chsB-pyrG	AGAACTAAATTACCGCCAGGCCGACCGGTCGCCTCAAACAATGCTCTTCA
MHG_234	Rev fus pyrG pr. chsB	AGCCAATCTTATTGAGCACCAAGCTGTCTGAGAGGAGGCACTGATGC
MHG_235	Fw prom. chsB	CTTGGTGCTCAATAAGATTGGC
MHG_236	Rev 5'utr chsB	GGTTAAACTGGTAGTATGTGCG
MHG_237	Fw fus 5'utr chs-GFPm	ATTCGCACATACTACCAGTTTAACCATGAGCAAGGGCGAGGAGCTCTTC

Code	Name	5’-3’ Sequence
MHG_238	Rev fus GFPm-GA	ACCGGCTCCAGCGCCTGCACCAGCTCCCTTGTAGAGCTCGTCCATGCCG
MHG_239	Fw fus ga-chsB sin atg	GAGCTGGTGCAGGCGCTGGAGCCGGTGCCGCCTACCACGGCTCTGGTCC
MHG_261	rev GAs	GGCACCGGCTCCAGCGCC
MHG_262	fw fus 5'utr chsB-mcherry	TCTATTCGCACATACTACCAGTTTAACCATGGTGAGCAAGGGCGAGGAG
MHG_282	Fw fus up chsB-riboBAf	GAACTAAATTACCGCCAGGCCGAAGAGGCCGTTCAGGAGTCTGG
MHG_283	Rev fus riboBAf-prom chsB	GCCAATCTTATTGAGCACCAAGCAGAACGTTTGCGCTGCAGAAC
IBP70	Fw AN2736 (vps53)_ext	GAATGTTTCCCAATTCCTGGAC
IBP71	Rv AN2736 (vps53)_ext	GAGAAGAACCTACATCCTTAGCG
IBP88	Fw upstream AN3015	GCATCCTGAAGAATTAATCTGTG
IBP89	Rv upstream AN3015	CATGTATTATACGAAAGGATCTGG
IBP90	Fw AN3015-pyrGAf	CAGATCCTTTCGTATAATACATGACCGGTCGCCTCAAACAATGCTC
IBP91	Rv AN3015-pyrGAf	GATCAACTCCAAATCAGCTCCGTCTGAGAGGAGGCACTGATG
IBP92	Fw downstream AN3015	GGAGCTGATTTGGAGTTGATC
IBP93	Rv downstream AN3015	CAAGATGGCTTATTCACCAATG
IBP103	Fw AN3015 externalNEW	CCAGCAGTCTTGCCTTCCTC
IBP104	Rv AN3015 externalNEW	GATCTTTCGTGCTGACAGTGC
IBP110	Fw ORF vps54-Stag	GAGAAGACACGGTCTGCCGTG
IBP111	Rv ORF vps54-Stag	GTGCTGGCCCTGTCCCTTGG
IBP112	Fw vps54-S-pyrG	GAGGCCAAGGGACAGGGCCAGCACGGAGCTGGTGCAGGCGCTGG
IBP113	Fw Vps54-S_EXT	CCACCACATTCTCCTGCAGTC
IBP114	Rv Vps54-S_EXT	CTTCTAGAACCAGCGGATGCG
IBP115	Fw ORF vps51-Stag	CACCCGCCTCCTCGCGAC
IBP116	Rv ORF vps51-Stag	ATCGGCTTCGTCCTCCTTTTGC
IBP117	Fw vps51-S-pyrG	GCAAAAGGAGGACGAAGCCGATGGAGCTGGTGCAGGCGCTGG
IBP118	Fw vps51-S-tag EXT	CATGATGTCTACCATCTCCTC
IBP121	Fw upstream vps53	CAGCGATGTGCTATGTTTGCG
IBP122	Rv upstream vps53	CATTAGTTCTCGGGAATTGACG
IBP123	Fw pyrGaf-vps53	CGTCAATTCCCGAGAACTAATGCCGGTCGCCTCAAACAATGC
IBP124	Rv pyrGaf-vps53	GCCCACCCACATCAGCCATCGTCTGAGAGGAGGCACTGATG

Code	Name	5’-3’ Sequence
IBP125	Fw downstream vps53	GATGGCTGATGTGGGTGGGC
IBP126	Rv downstream vps53	GGAACAGCCAGCTGAAGGCG
MP362	Fw 5UTR Vps52	CCTTGGCGTGACCGTGCTTACC
MP363	Rev 5UTR Vps52	GTTGGAGCGCGAAGGATATTAG
MP364	Fw join 5UTR Vps52 pyrGAf	TCCTCCTAATATCCTTCGCGCTCCAACGTAACGGCCGCCAGTGTGCTG
MP365	Rev join 3UTR Vps52- pyrGAf	GTGCTATGAATAGCACCGGTGTGCATTCTGTCTGAGAGGAGGCACTGATG
MP366	Fw 3UTR Vps52	AGAATGCACACCGGTGCTATTC
MP367	Rev 3UTR Vps52	CGTTACGTCCCATTGCTCTTC
MP419	Fw 5UTR Vps54	CAGTAAGTCTTCGCCACTCAGCC
MP420	Rev 5UTR Vps54	CTCGCCGTCAAACTGGCGACTC
MP421	Fw join 5UTR Vps54-pyrGAf	GCTGTTGAGTCGCCAGTTTGACGGCGAGGTAACGGCCGCCAGTGTGCTG
MP422	Rev join 3UTR Vps54-pyrGAf	GCGCACACATGGTGATGATTATAGGTACGTCTGAGAGGAGGCACTGATG
MP423	Fw 3UTR Vps54	GTACCTATAATCATCACCATGTGTG
MP424	Rev 3UTR Vps54	CGCTCTCAGACCAATGACGCC
